# Impact of N-acetyltransferase 10 on macrophage activation and inflammation-induced cardiac dysfunction

**DOI:** 10.1038/s41419-025-07796-6

**Published:** 2025-07-01

**Authors:** Zilong Xiao, Xiang Wei, Peng Li, Ruizhen Chen, Ziqing Yu, Yixiu Liang, Yangang Su, Junbo Ge

**Affiliations:** 1 Department of Cardiology, Zhongshan Hospital, Fudan University, Shanghai, China; 2 Shanghai Institute of Cardiovascular Diseases, Shanghai, China; 3 National Clinical Research Center for Interventional Medicine, Shanghai, China; 4 Institute of Cardiovascular Physiology and Pathophysiology, Biomedical Center, Ludwig-Maximilians-Universität München, Planegg-Martinsried, Germany; 5 Department of Anesthesiology, Zhongshan Hospital, Fudan University, Shanghai, China; 6 National Heart and Lung Institute, Imperial College London, Hammersmith Hospital, Du Cane Road, London, UK

**Keywords:** Cytokines, Cell signalling

## Abstract

Inflammation-induced cardiac dysfunction, driven by an abnormal immune response, significantly contributes to sepsis-related mortality. Controlling excessive pro-inflammatory cytokine production by immune cells remains a significant challenge. This study investigated the role of N-acetyltransferase 10 (NAT10) in macrophage activation and its contribution to inflammation-induced cardiac dysfunction. Using bone marrow-derived macrophages and an endotoxemia mouse model, we found that NAT10 is significantly upregulated in response to lipopolysaccharide (LPS) due to the deubiquitinating enzyme USP39, which stabilizes the NAT10 protein. ac4C RNA sequencing identified ETS2 as a direct target of NAT10, where the ac4C modification enhanced ETS2 mRNA stability and translation, promoting a pro-inflammatory phenotype in macrophages. NAT10 deficiency reduces LPS-induced macrophage activation and cytokine production, improving cardiac function in mice. Pharmacological inhibition of NAT10 using remodelin produced similar protective effects. Our findings reveal a novel post-transcriptional pathway and highlight the therapeutic potential of targeting NAT10 to mitigate inflammation-induced cardiac injury in endotoxemia

## Introduction

Endotoxemia (ETM) or sepsis poses a severe issue in modern critical care medicine and can cause multiple organ failure and death [1]. Bacterial endotoxins, or lipopolysaccharides (LPS), released by gram-negative bacteria cause severe inflammation during ETM [2]. Clinical management of sepsis traditionally involves using antibiotics and volume replacement [3]. Nevertheless, approximately 40–50% of patients with ETM still experience cardiac dysfunction and heart failure due to the excessive inflammatory response and absence of specific therapies to improve cardiac function [4, 5]. Consequently, interventions aimed at modulating inflammation hold promise for preventing ETM-induced cardiac dysfunction.

Myeloid cells directly detect bacterial endotoxins, triggering the activation of transcriptional and epigenetic networks that facilitate the transition from a steady state to an inflammatory programming state [6]. Reportedly, macrophages exert dual effects. Although they are crucial for combating infections, they also secrete excessive cytokines [7], resulting in a detrimental systemic inflammatory response syndrome and subsequent organ dysfunction [8]. Uncontrolled inflammation exacerbates cardiac injury by hindering cardiomyocyte contractility and increasing cardiomyocyte death [9]. Conversely, appropriate modulation of macrophage function can mitigate inflammatory infiltration and promote cardiac function [3, 4]. Therefore, the well-orchestrated mechanism of macrophage activation during sepsis requires further investigation in biology and medicine.

The post-transcriptional RNA modification is a critical part of epigenetics, with mRNA undergoing approximately 170 types of post-transcriptional modifications, such as N6-methyladenosine (m6A), N1-methyladenosine, and N4-acetylcysteine (ac4C) [10–12]. These mRNA modifications are controlled by highly conserved enzymatic complexes, whose functions can be significantly changed during the disease [11, 13]. ac4C, a newly discovered RNA modification, and its “writer” N-acetyltransferase 10 (NAT10) are essential for mRNA translation [14]. ac4C is prevalent in the human transcriptome at physiologically relevant levels [14, 15], predominantly within coding sequences (CDS), promoting translation efficiency through enrichment of the C base at the unstable position [14]. NAT10, a human enzyme, catalyzes acetylation and binds to RNA [16]. Its disruption ablates ac4C modifications at specific RNA sites, and studies have shown that ac4C levels and NAT10 expression are associated with the development of various diseases, including cancers, autoimmune, cardiovascular, and inflammatory diseases [17, 18]. While some studies have explored the roles of ac4C and NAT10 in inflammatory diseases, the underlying mechanism of ac4C modifications in macrophages and ETM-induced myocardial dysfunction remains underexplored.

This study investigates the role of NAT10 in macrophage activation and its contribution to inflammation-induced cardiac dysfunction. We observed significant alterations in ac4C and NAT10 protein levels in LPS-stimulated macrophages. Using ac4C sequencing, ribosome sequencing, and transcriptome analysis, we identified transcription factor Ets2 as a direct NAT10 target. Our findings provide further insight into the roles of Ets2 and NAT10 in macrophage function, revealing a post-transcriptional mechanism that regulates the inflammatory response in endotoxemia.

## Results

### NAT10 is upregulated in macrophages upon LPS challenge

**Fig. 1:**
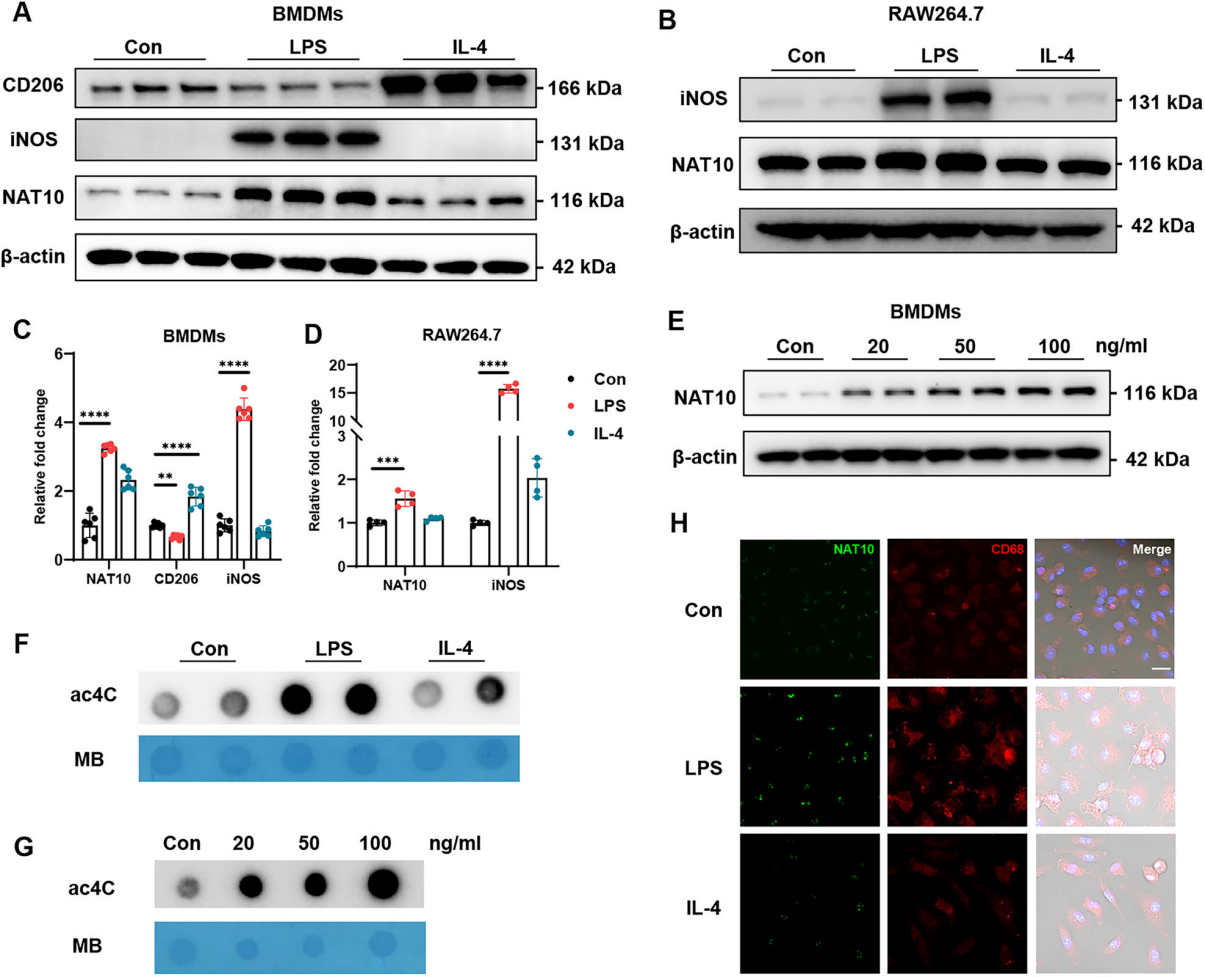
NAT10 protein levels and ac4C modification levels are upregulated in BMDMs upon LPS treatment. **A** Western blot analysis of NAT10 protein levels in BMDMs treated with PBS, LPS, or IL-4 for 24 h. **B** Western blot analysis of NAT10 protein levels in RAW264.7 cells treated with PBS, LPS, or IL-4 for 24 h. **C** Quantitative analysis of NAT10 protein levels in BMDMs and RAW264.7 cells (*n* = 6 per group). **D** Quantitative analysis of NAT10 protein levels in RAW264.7 cells (*n* = 4 per group). **E** Western blot analysis of NAT10 protein levels in BMDMs treated with different doses of LPS. **F** Dot blot assay showing ac4C modification levels in BMDMs treated with PBS, LPS, or IL-4 for 24 h. **G** Dot blot assay showing ac4C modification levels in BMDMs treated with different doses of LPS. **H** Immunofluorescence staining showing the localization of NAT10 in BMDMs (scale bar = 10 μm). Data are shown as mean ± SD. Statistical analyses were performed using the One-way two-sided ANOVA.

Given the remarkable changes in the transcriptome and post-transcriptome of macrophages after activation [19], we hypothesized that ac4C modification may be vital for macrophage function. To test this, bone marrow-derived macrophages (BMDMs) were treated with two well-characterized ligands, LPS and IL-4. NAT10 protein levels were significantly higher in LPS-induced BMDMs than in IL-4-induced BMDMs (Fig. 1A–C). The increase in NAT10 protein level was dose-dependent (Fig. 1D). Similar findings were obtained in RAW264.7 cells (Fig. S1A). Notably, NAT10 protein levels in BMDMs peaked 24 h after LPS stimulation (Fig. S1B). To investigate the impact of changes in NAT10 expression on ac4C levels in macrophages, we conducted a dot blot analysis to measure ac4C modifications in RNA extracted from LPS- or IL-4-treated macrophages. The ac4C levels remained relatively unchanged after IL-4 stimulation but increased sharply after LPS treatment (Fig. 1E). Immunofluorescence analysis confirmed the nuclear localization of NAT10 (Fig. 1F). Thereafter, the mechanisms underlying the robust alteration in NAT10 expression in macrophages were explored. qPCR analysis indicated no significant changes in Nat10 mRNA levels following macrophage activation (Fig. S1C). Sequencing data from the GENE EXPRESSION OMNIBUS (GEO) database further supported this hypothesis (Fig. S1D). These findings suggested that NAT10 is post-transcriptionally regulated in response to macrophage activation.

### LPS stabilizes NAT10 by promoting NAT10-USP39 interaction and reducing proteasomal degradation

**Fig. 2:**
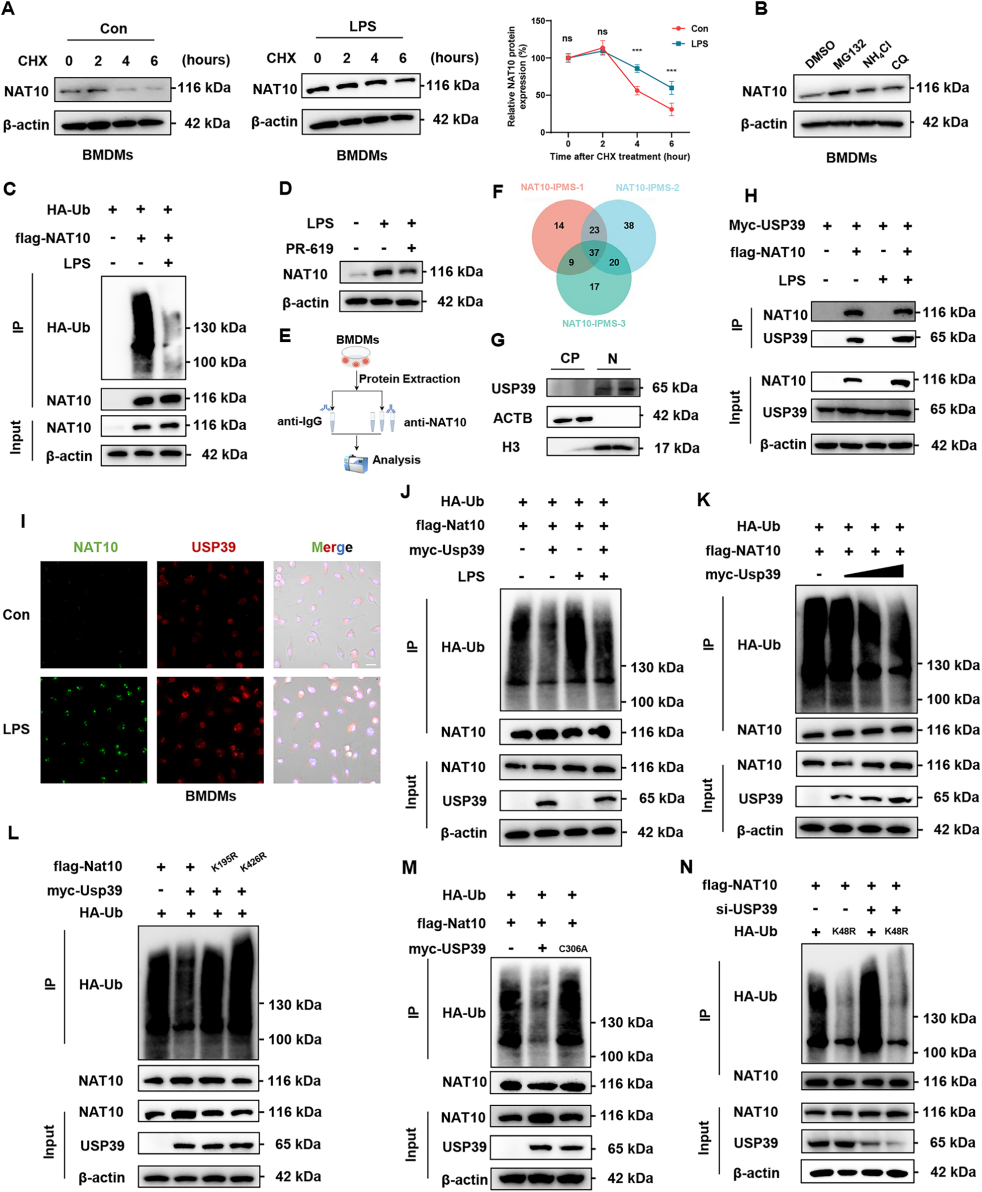
USP39 interacts with NAT10 and inhibits its degradation by reducing K48-linked polyubiquitination. **A** Protein decay assays for NAT10 in LPS-treated BMDMs at different time points following CHX treatment (*n* = 3 independent experiments). **B** Western blot analysis of NAT10 protein expression in BMDMs treated with MG132 (10 μM), NH₄Cl (25 mM), or chloroquine (25 μM) for 6 h. **C** Ubiquitination plasmids (Ub) were transfected into RAW264.7 cells treated with PBS or LPS, and NAT10 protein was immunoprecipitated. RAW264.7 cells were treated with MG132 (25 μM) for 8 h before sample collection, and NAT10 ubiquitination levels were detected by Western blot. **D** Western blot analysis of NAT10 protein levels in BMDMs treated with LPS and pan-deubiquitinase inhibitor PR-619 (25 μM) for 8 h. **E**, **F** A Venn diagram reveals that USP39 is the only deubiquitinase identified at the intersection of the three IP-MS groups. Potential deubiquitinating enzymes (DUBs) interacting with NAT10 were identified by mass spectrometry in BMDMs. **G** Western blot analysis of cytoplasmic and nuclear fractions of BMDMs after LPS treatment shows that USP39 is predominantly localized in the nucleus. **H** RAW264.7 cells were transfected with Myc-tagged USP39 and Flag-tagged NAT10, followed by treatment with PBS or LPS for 24 h. Cell lysates were immunoprecipitated using an anti-Flag antibody, and both the immunoprecipitated proteins and input were analyzed by Western blot to assess protein interactions and expression levels. **I** Colocalization of USP39 and NAT10 was examined by confocal microscopy (Scale bar = 10 μm). **J** RAW264.7 cells were transfected with USP39, NAT10, and HA-UB plasmids, and ubiquitinated NAT10 protein was immunoprecipitated. Ubiquitinated RAW264.7 cells were treated with MG132 (25 μM) for 8 h before sample collection, and NAT10 ubiquitination levels were detected by Western blot. **K** 293 T cells transfected with the indicated constructs were treated with MG132 for 8 h before collection. Whole-cell lysates were subjected to immunoprecipitation with a Flag antibody and Western blot with an anti-HA antibody to detect NAT10 ubiquitination levels. **L** USP39-mediated deubiquitination of NAT10 was significantly reduced in 293 T cells transfected with NAT10-K195R or NAT10-K426R mutants. **M** Effect of USP39 and the catalytically inactive USP39-C306A mutant on the ubiquitination of NAT10. **N** USP39 regulates K48-linked ubiquitination of NAT10. Data are shown as mean ± SD. Statistical analyses were performed using the One-way two-sided ANOVA.

As protein degradation is crucial for regulating protein levels [20], we used a cycloheximide (CHX) chase assay to examine the stability of NAT10. The results demonstrated that the half-life of NAT10 was significantly prolonged after LPS activation (Fig. 2A). We found that NAT10 degradation occurred via the proteasome pathway, not autophagy or lysosomal pathways (Fig. 2B). Additionally, NAT10 ubiquitination was significantly reduced in LPS-treated macrophages (Fig. 2C). To investigate whether ubiquitination or deubiquitination enzymes affect NAT10 stability, we used PR-619, a broad-spectrum deubiquitinating enzyme inhibitor. Notably, PR-619 reversed the LPS-induced increase in NAT10 protein levels, indicating that deubiquitination is a key regulatory mechanism for NAT10 stability (Fig. 2D). To identify potential deubiquitinases, we conducted immunoprecipitation (IP)/mass spectrometry in LPS-treated BMDMs to analyze the NAT10 interactome and identified USP39 as the only candidate that functions as a deubiquitinase (Fig. 2E, F). To validate NAT10 as a direct substrate of USP39, we demonstrated that USP39 colocalizes with NAT10, with both proteins predominantly expressed in the nucleus (Fig. 2G–I). Moreover, in USP39 knockdown RAW264.7 cells, NAT10 expression was significantly reduced, underscoring the role of USP39 in maintaining NAT10 levels (Fig. S2C–E). The interaction between NAT10 and USP39 was further confirmed by Co-IP (Fig. 2H and Fig. S2A, B). USP39 overexpression decreased NAT10 ubiquitination in RAW264.7 and HEK293T cells (Fig. 2J, K). To investigate whether the deubiquitinase activity of USP39 is responsible for NAT10 stability, we generated a USP39 mutant (C306A) with lost deubiquitinase activity [21]. As expected, the C306A mutation failed to reduce NAT10 ubiquitination in HEK293T cells (Fig. 2M). Additionally, K195 and K426 were identified as key ubiquitination sites in NAT10, as previously reported [17]. We introduced lysine-to-arginine point mutations at these sites to confirm the role of USP39-mediated deubiquitination in the regulation of NAT10 stability (Fig. 2L). Among the various ubiquitination site modifications, K-48 is often considered to be highly correlated with protein degradation [22, 23]. USP39 specifically removed the K48-linked ubiquitin chains from NAT10, enhancing its stability (Fig. 2N).

### NAT10 Deficiency Suppresses pro-inflammatory macrophage activation

**Fig. 3:**
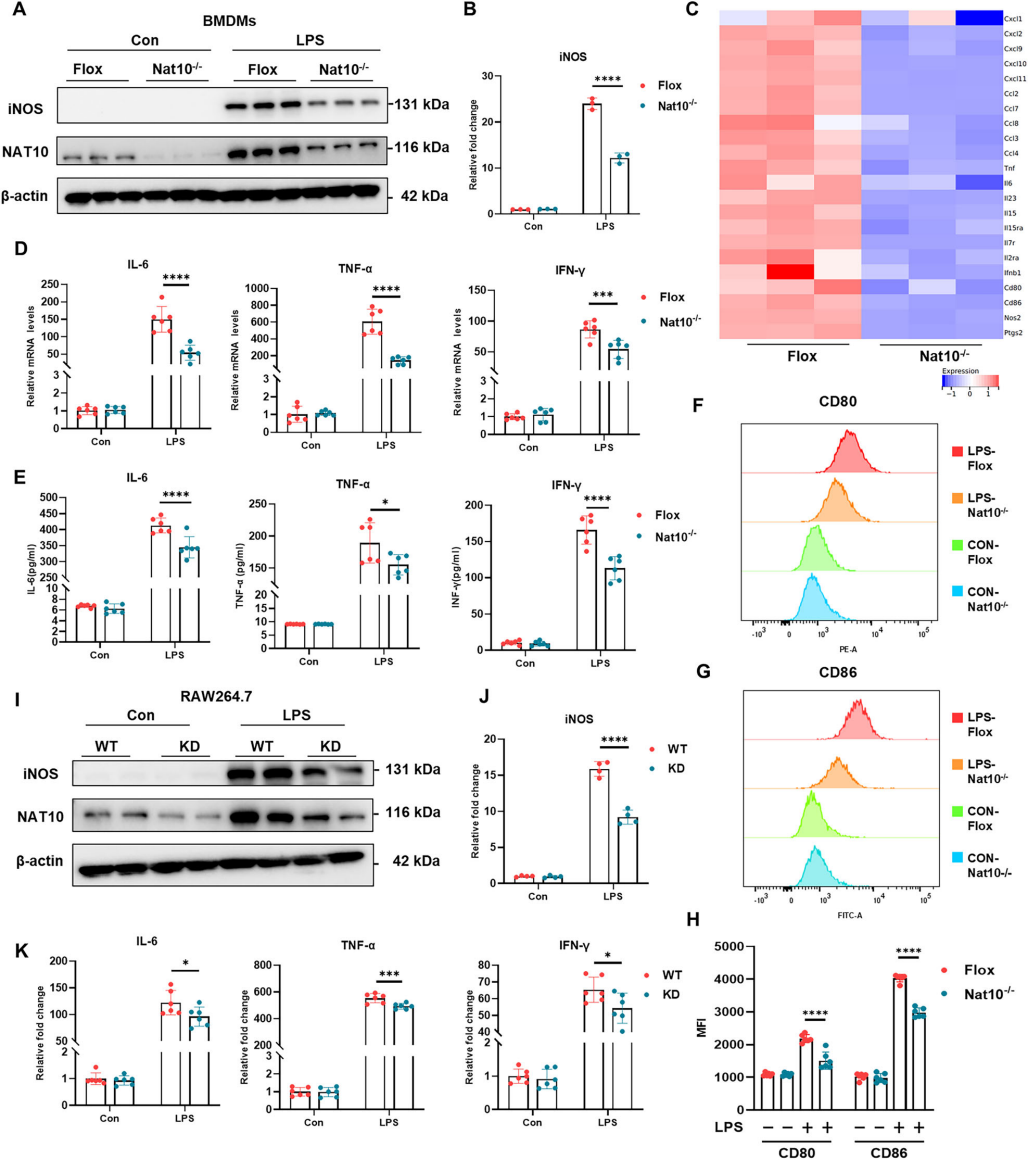
NAT10 depletion inhibits LPS-induced macrophage activation. **A** Immunoblot analysis of INOS in whole cell lysates of Flox or Nat10-/- BMDMs stimulated with LPS (100 ng/mL) for 24 h. **B** Quantitative analysis of INOS protein levels in BMDMs(*n* = 3 per group). **C** Heatmap showing the expression levels of inflammation-related genes from RNA sequencing of Flox and NAT10-/- BMDM cells following LPS treatment. **D** Real-time PCR analysis of mRNA expression of cytokines in Flox or Nat10-/- BMDMs with or without LPS treatment(*n* = 6 per group). **E** ELISA analysis of cytokine secretion in the culture medium of Flox or Nat10-/- BMDMs with or without LPS treatment(*n* = 6 per group). **F**, **G**, **H** Flow cytometry analysis of macrophage surface markers CD80 and CD86 expression in Flox or Nat10-/- BMDMs with or without LPS treatment. **I** Immunoblot analysis of INOS in whole cell lysates of WT or Nat10 + /- RAW264.7 cells stimulated with LPS (100 ng/mL) for 24 h. **J** Quantitative analysis of INOS protein levels in RAW264.7 cells (*n* = 4 per group). **K** Real-time PCR analysis of mRNA expression of cytokines in WT or Nat10 + /- RAW264.7 cells with or without LPS treatment. Data are shown as mean ± SD. Statistical analyses were performed using the Two-way two-sided ANOVA.

Toll-like receptor activation in macrophages triggers extensive cytokine release, exacerbating the inflammatory response [24]. To investigate the role of NAT10 in macrophages, we generated myeloid-specific Nat10 knockout mice and isolated BMDMs for subsequent experiments (Fig. S3A–C). Notably, inducible nitric oxide synthase (iNOS), a typical biomarker of M1-like macrophages, was significantly downregulated in LPS-treated Nat10^-/-^ macrophages (Fig. 3A, B). Transcriptomic analysis identified numerous differentially expressed genes related to inflammatory cytokines and chemokines between the Flox and NAT10^-/-^ groups (Fig. 3C). Enrichment analysis revealed several pathways associated with inflammatory activation (Fig. S4A–D). RT-qPCR confirmed changes in key inflammatory factors, and ELISA demonstrated reduced cytokine secretion in the culture media (Fig. 3D–E). A similar trend was observed with the decreased expression of the cell surface proteins CD80 and CD86 (Fig. 3F–H). Additionally, treatment of BMDMs with remodelin, a well-known Nat10 inhibitor, diminished the inflammatory response to LPS (Fig. S5A–E). To further validate these findings, we conducted parallel experiments using RAW264.7 cells. NAT10-knockdown RAW264.7 cells exhibited lower levels of iNOS protein and reduced mRNA levels of IL-6, TNF-α, and IFN-γ (Fig. 3I–H). Conversely, Nat10 overexpression exacerbated mRNA levels and secretion of IL-6, TNF-α, and IFN-γ in RAW264.7 cells (Fig. S6A–F). Collectively, these findings suggested that NAT10 plays a critical role in LPS-mediated macrophage activation.

### NAT10 mediates macrophage activation in an ac4C-dependent manner

**Fig. 4:**
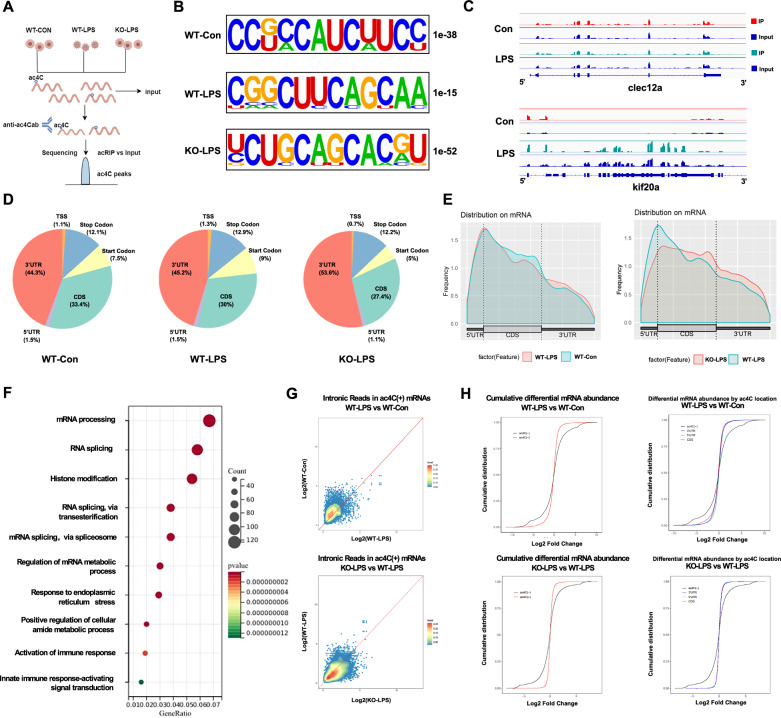
NAT10 (N-acetyltransferase 10)-mediated N4-acetylcysteine (ac4C) RNA acetylation changes are associated with LPS-induced inflammatory activation of macrophages. **A** Schematic of the acRIP-seq procedure. **B** Predominant motif identified within ac4C-seq peaks among WT-CON, WT-LPS, and NAT10-/- -LPS groups. **C** Visualization of ac4C modification on the target gene kif20a and the non-acetylated gene clec12a using the IGV browser. **D** Genomic distributions of ac4C peaks in WT-CON, WT-LPS, and NAT10-/- -LPS groups. **E** Distribution of acetylated positions in mRNA between WT-LPS vs. WT-CON and NAT10-/- -LPS vs. WT-LPS groups. **F** GO biological process analysis for hyperacetylated and hypoacetylated transcripts between WT-LPS and WT-CON groups. **G** Normalized exonic reads from ac4C(+) transcripts in WT-LPS vs. WT-CON and NAT10-/- -LPS vs. WT-LPS groups. **H** Left: CDF plot depicting differential expression of ac4C (−) or ac4C (+) transcripts in WT-LPS vs. WT-CON and NAT10-/- -LPS vs. WT-LPS groups. Right: CDF plot depicting differential expression genes in WT-LPS vs. WT-CON and NAT10-/- -LPS vs. WT-LPS groups for ac4C (−) and ac4C (+) transcripts with peaks occurring within the CDS, 5’UTR, or 3’UTR.

To determine whether Nat10 contributes to macrophage activation through ac4C modification, we performed ac4C-specific RNA sequencing analyses of wild-type BMDMs, LPS-stimulated wild-type BMDMs, and LPS-stimulated Nat10^-/-^ BMDMs (Fig. 4A). Most ac4C peaks were located within the distinct CXXCXX motif, which is consistent with previous studies [14] (Fig. 4B). Clec12a and Kif20a served as non-enriched controls and highly enriched ac4C targets, respectively (Fig. 4C). All the samples contained numerous ac4C modified sites, aligning with previous reports [25] (Fig. S7A). Most ac4C peaks in BMDMs were enriched in the 3′UTRs (Fig. 4D). Most ac4C-containing transcripts had modifications in the 5′UTR, 3′UTR, or CDS, with only a few transcripts carrying modifications simultaneously at all three sites (Fig. S7D). To assess the role of NAT10-induced ac4C modification in LPS-stimulated macrophages, we conducted an enrichment analysis of hyperacetylated and hypoacetylated transcripts in the WT-LPS and WT-CON groups. Gene ontology (GO) biological process (BP) and KEGG analyses revealed that the top-ranked enriched pathways were predominantly involved in RNA post-transcriptional modification and inflammatory activation (Fig. 4F, Fig. S7E). Similar results were observed in the enrichment analysis of differentially hyperacetylated and hypoacetylated transcripts in KO-LPS versus WT-LPS (Fig. S7F–G). This suggested that Nat10 regulates macrophage inflammatory activation via ac4C modifications.

To determine whether the ac4C modification drives changes by influencing mRNA abundance or transcription, we performed intronic signal analyses and cumulative distribution function (CDF) analysis across the three groups. First, when normalized to intronic reads, we observed that the overall transcription of ac4C(+) mRNAs was not significantly altered compared with WT-CON vs. WT-LPS and WT-LPS vs. KO-LPS (Fig. 4G). CDF plots demonstrated that the distributions of ac4C(+) and ac4C(-) gene differences were balanced when comparing WT-LPS and WT-CON macrophages. Similar results were observed when the WT-LPS and KO-LPS groups were compared (Fig. 4H). Therefore, we postulated that Nat10-regulated ac4C modification influences macrophage inflammatory activation by altering the expression of key genes rather than changing the overall mRNA abundance.

### NAT10 promotes Ets2 mRNA stability and translation efficiency

**Fig. 5:**
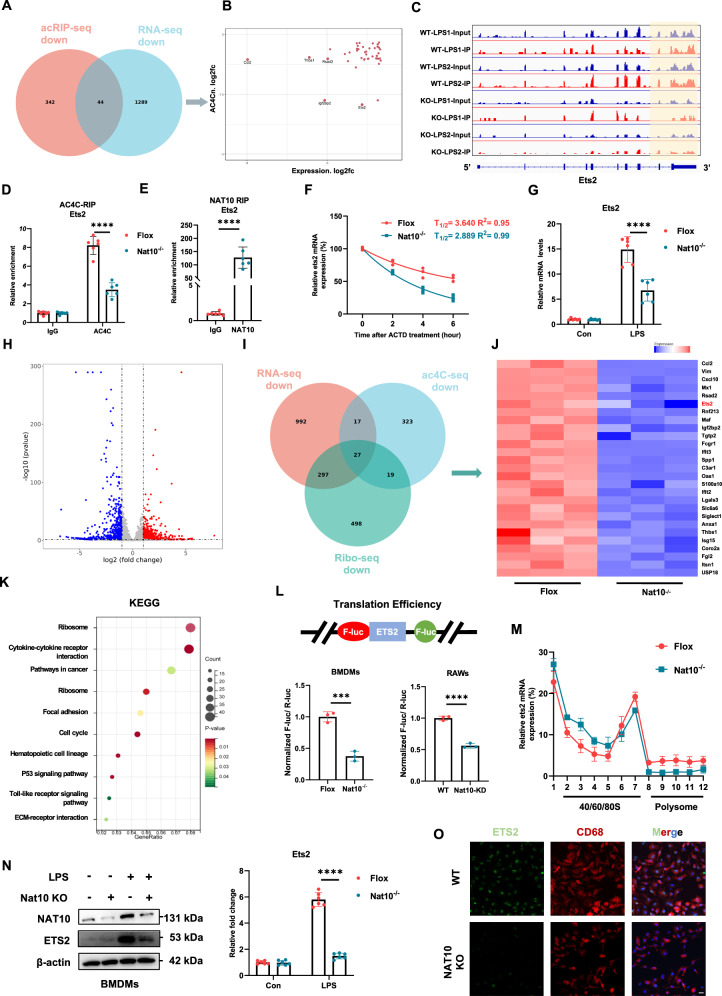
NAT10-mediated ac4C RNA acetylation promotes Ets2 mRNA stability and translation efficiency. **A** Venn diagram showing the downstream target genes regulated by NAT10 via ac4C modification. Left: Hypoacetylated genes after NAT10 knockout (acRIP-seq); Right: Downregulated genes after NAT10 knockout (RNA-seq, *P* < 0.05, log2FoldChange < -1). **B** Fold changes in transcript levels (RNA-seq) and ac4C modification levels of 44 downstream targets. **C** Visualization of ac4C peaks in Ets2 across different groups using IGV software. **D** RT-qPCR detection of the relative enrichment of Ets2 mRNA in acRIP products from BMDMs. **E** RT-qPCR detection of the relative enrichment of Ets2 mRNA in NAT10 RIP products from BMDMs. **F** Decay curves for Ets2 mRNA in Flox and Nat10-/- BMDMs. **G** RT-qPCR detection of Ets2 mRNA expression in BMDMs after Nat10 knockout. **H** Volcano plot of differentially expressed genes ( | log2FC | > 1 and FDR < 0.05) at the translation level in LPS-treated BMDMs after Nat10 knockout. **I** Venn diagram showing the overlap of target genes with changes in ac4C acetylation, transcription, and translation levels after NAT10 knockout. **J** Heatmap of translation efficiency (Ribo-seq) for the overlapping 27 target genes., expressed as Z-score in relation to median. **K** KEGG analysis of differentially expressed genes (DEGs) in Ribo-seq. **L** Nat10-depleted or control cells were transfected with lentivirus containing pmirGLO-Ets2 reporter for 24 h, and Ets2 translation efficiency was defined as reporter protein production (F-luc/R-luc) divided by mRNA abundance. **M** Relative mRNA distribution of Ets2 in ribosome fractions analyzed by qRT-PCR in BMDMs. **N** Western blot analysis and densitometric quantification of ETS2 protein in Flox and Nat10-/- BMDMs. **O** Immunofluorescence analysis showing changes in ETS2 localization and expression. (scale bar = 10 um). Data are shown as mean ± SD. Statistical analyses were performed using the student *t*-test (**E**, **L**) and two-way two-sided ANOVA (**D**, **G**, **N**).

To identify the key genes, we performed a combined analysis of ac4C-seq and RNA-seq from the KO-LPS and WT-LPS groups. The intersection of the Venn diagrams revealed 44 differentially expressed genes in RNA seq and ac4C-specific RNA seq results (Fig. 5A). We used a scatter plot to illustrate the differences in the ac4C modification levels and RNA abundance for these expressed genes (Fig. 5B). Among the overlapping genes, Ets2 exhibited the most significant changes in ac4C modification levels, and recent studies underscored its strong association with macrophage inflammatory activation [26] (Fig. 5B). Therefore, we focused on Ets2. Visualization analysis of the ac4C peaks using Integrative Genomics Viewer (IGV) software revealed a reduction in ac4C peak signals following Nat10 suppression (Fig. 5C). The ac4C-RIP-qPCR results confirmed that the ac4C modification level of Ets2 was significantly downregulated following Nat10 knockout (Fig. 5D). To determine whether NAT10 directly binds to Ets2 mRNA and mediates its acetylation, the binding sites between NAT10 and Ets2 mRNA were predicted using the catRAPID algorithm (Fig. S8A). NAT10-RIP-qPCR and RNA pull-down assays further confirmed that NAT10 directly binds to the Ets2 transcript (Fig. 5E, Fig. S9C). As the ac4C modification has been reported to promote mRNA stability [14], we performed an RNA stability assay and confirmed that Ets2 mRNA stability was significantly reduced after NAT10 depletion (Fig. 5F). RT-qPCR analysis demonstrated a notable reduction in Ets2 mRNA levels in Nat10^-/-^ BMDMs (Fig. 5G).

Considering that Nat10 affects the translation efficiency of its substrates [14], we performed Ribo-seq to assess the ribosomal loading of mRNA (KO-LPS vs. WT-LPS) (Fig. 5H). GO BP and KEGG analyses showed the functional enrichment of transcripts with differential translation efficiencies (Fig. 5K, Fig. S9A). Comparing translation efficiency in BMDMs with or without Nat10 deletion showed no evidence that NAT10-mediated mRNA ac4C modification enhanced global translation efficiency in BMDMs (Fig. S9B). These results further led us to search for the crucial targets of Nat10. We intersected data from Ribo-seq (downregulated genes), ac4C-seq (hypoacetylated genes), and RNA-seq (downregulated genes) and analyzed them in a Venn diagram (Fig. 5I). Ets2 (log2FoldChange = *−*1.2) remained among the 27 intersecting genes (Fig. 5J). To validate whether Ets2 translation efficiency was modulated by Nat10, dual-luciferase assays, and polysome profiling were performed. We constructed a pmirGLO-Ets2 luciferase reporter by ligating the Ets2 CDS into multiple cloning sites. The translation efficiency of Ets2 was lower in NAT10-deficient cells than in controls (Fig. 5L). Additionally, polysome profiling showed a significant reduction in Ets2 mRNAs within translation-active polysomes ( > 80S) in NAT10^-/-^ cells compared with control cells (Fig. 5M). Furthermore, ETS2 protein levels were significantly decreased in NAT10-depleted BMDMs and RAW264.7 cells (Fig. 5N, O, Fig. S9D), while NAT10 overexpression increased ETS2 levels in RAW264.7 cells (Fig. S9E). Collectively, these results suggest that NAT10 promotes Ets2 mRNA stability and enhances its translation efficiency.

### The NAT10/ETS2 axis is involved in the mechanism of macrophage activation

**Fig. 6:**
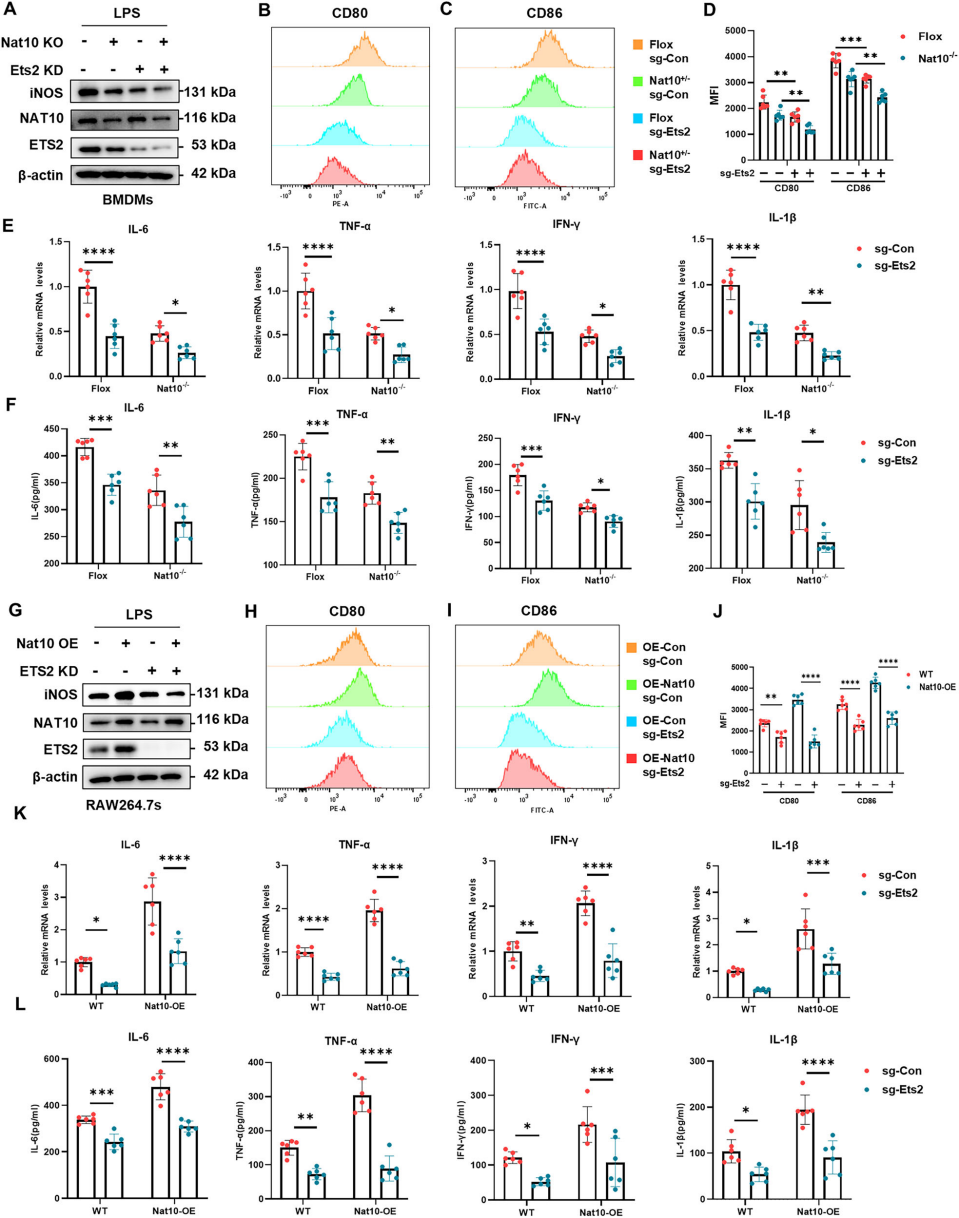
The NAT10/ETS2 axis is involved in the mechanism of LPS-induced macrophage activation. **A** Western blot detection of iNOS, NAT10, and ETS2 protein levels in BMDMs. Flox and Nat10-/- BMDMs were transfected with or without Ets2 lentivirus. **B–D** Flow cytometry analysis of macrophage surface markers CD80 and CD86 expression in BMDMs of each group(*n* = 6 per group). **E** Real-time PCR analysis of mRNA expression of cytokines in BMDMs of each group(*n* = 6 per group). **F** ELISA analysis of cytokine secretion in the culture medium of BMDMs of each group(*n* = 6 per group). **G** Western blot detection of iNOS, NAT10, and ETS2 protein levels in RAW264.7 cells. WT and Nat10 stable overexpressing RAW264.7 cells were transfected with or without Ets2 lentivirus. **H–J** Flow cytometry analysis of macrophage surface markers CD80 and CD86 expression in RAW264.7 cells of each group(*n* = 6 per group). **K** Real-time PCR analysis of mRNA expression of cytokines in RAW264.7 cells of each group(*n* = 6 per group). **L** ELISA analysis of cytokine secretion in the culture medium of RAW264.7 cells of each group (*n* = 6 per group). Data are shown as mean ± SD. Statistical analyses were performed using two-way two-sided ANOVA.

Our findings revealed that Nat10 deficiency attenuated pro-inflammatory activation, alongside reduced Ets2 levels in BMDMs, due to decreased ac4C modification. This suggests that the NAT10/ETS2 axis plays a central role in the LPS-induced pro-inflammatory response in macrophages. To validate this, Nat10^-/-^ BMDMs and WT BMDMs were transduced with Ets2 lentivirus or empty vector, followed by an assessment of inflammatory cytokine markers. Western blot and flow cytometry analyses showed that iNOS protein expression and M1 surface marker levels were lower in Ets2-knockdown macrophages than in wild-type macrophages. The lowest levels of iNOS protein and M1 surface markers were observed in Nat10-deficient macrophages following Ets2 knockdown (Fig. 6A–D). The mRNA expression and secretion of pro-inflammatory cytokines, as detected by qPCR and ELISA, aligned with these findings (Fig. 6E, F). Similar experiments and analyses were conducted in RAW264.7 cells, yielding the same results (Fig. S10A–F). To further elucidate the role of the NAT10/ETS2 axis, wild-type and Nat10-overexpressing RAW264.7, cells were transduced with Ets2 lentivirus or empty control, followed by an assessment of inflammatory cytokine markers. As expected, Ets2 knockdown partly mitigated Nat10 overexpression-induced exacerbation of macrophage activation, as evidenced by decreased levels of inflammatory markers, cytokine mRNAs, and secreted cytokines (Fig. 6G–L). Collectively, these results confirm that the NAT10/ETS2 axis regulates the inflammatory state in macrophages stimulated with LPS.

### Myeloid-Nat10 depletion ameliorates ETM-induced myocardial dysfunction

**Fig. 7:**
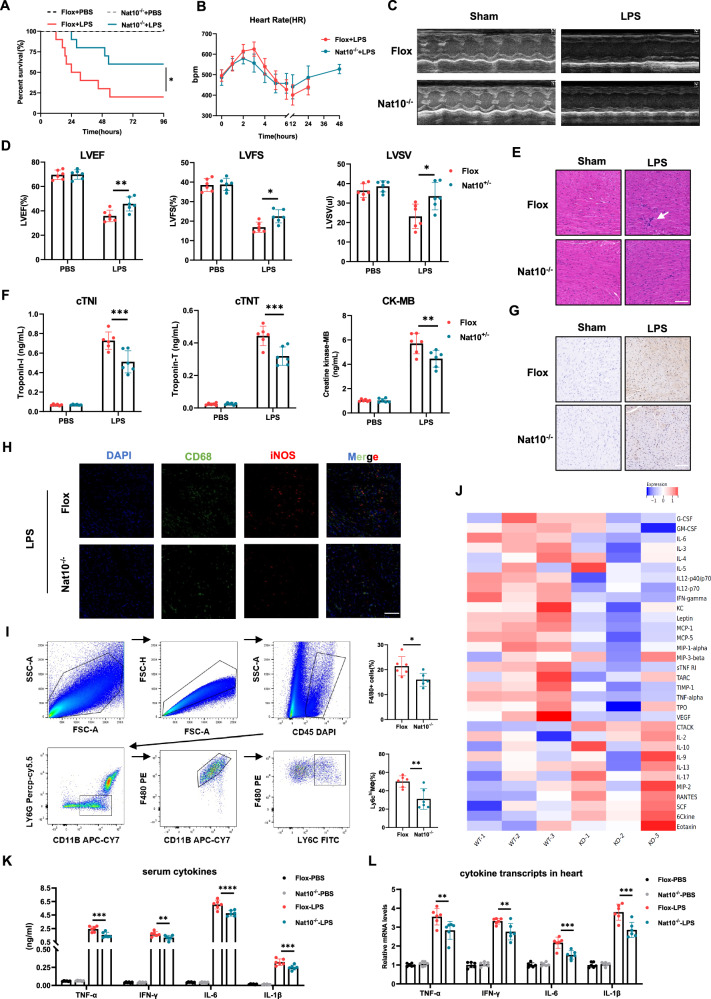
Myeloid-specific NAT10 depletion protects the heart during endotoxemia. **A** Kaplan-Meier survival curves of Flox and Nat10-/- mice (*n* = 10 male mice per group). **B** Mean heart rate of LPS-challenged Flox and Nat10-/- mice over 48 h (*n* = 10 male mice per group). **C**, **D** Representative M-mode echocardiographic images and statistical analysis of Left Ventricular Ejection Fraction, Fractional Shortening, and Stroke Volume in Flox and Nat10-/- mice 12 h after LPS challenge (*n* = 6 male mice per group). **E** Representative photomicrographs of ventricular tissues stained with hematoxylin and eosin (H&E). (scale bar = 100 um). **F** Serum levels of cardiac troponin-T, troponin-I, and creatine kinase-MB in LPS-challenged mice. **G** Representative immunohistochemical staining of CD45 in left ventricular myocardium. (scale bar = 100 um). **H** Representative immunofluorescent staining images of CD68 (green) and iNOS (red) in hearts of Flox and Nat10-/- mice during endotoxemia. (scale bar = 100 um). **I** Flow cytometry gating strategy and corresponding quantification showing the ratio of CD11B + + F4/80 + + macrophages and F4/80 + + Ly6C+high macrophages in each group (*n* = 6). **J** Heatmap of plasma cytokine changes in Flox and Nat10-/- mice during endotoxemia, expressed as Z-scores relative to the median. **K** ELISA analysis of cytokine levels in serum. **L** Real-time PCR analysis of cytokine expression in heart tissue (*n* = 6 per group). Data are shown as mean ± SD. Statistical analyses were performed using the Log-rank test (**A**) and two-way two-sided ANOVA (**D**, **F**, **H**, **K**, **L**).

To investigate the role of NAT10 in macrophage activity in vivo, we used an ETM mouse model induced by LPS peritoneal injection. qPCR analysis showed that Nat10 transcript levels remained unchanged, whereas Ets2 mRNA levels sharply increased in mouse blood mononuclear cells and heart tissue 12 h after LPS injection, which was consistent with the results from the GEO database (Fig.S11A–C). Immunofluorescence staining of heart tissue indicated macrophage infiltration into the heart, accompanied by increased NAT10 and ETS2 protein levels (Fig. S11D–F). Survival curve comparisons between Flox and Nat10^-/-^ mice subjected to LPS showed that Nat10^-/-^ mice had a higher survival rate than Flox controls (Fig. 7A). Monitoring of heart rate (HR) over 48 h revealed that Nat10 depletion inhibited sharp HR fluctuations (Fig. 7B). Echocardiographic parameters, including Left Ventricular Ejection Fraction (LVEF), Left Ventricular Fractional Shortening (LVES), and Left Ventricular Stroke Volume (LVSV), indicated improved cardiac function in Nat10^-/-^ mice 12 h after LPS treatment (Fig. 7C, D). ELISA analysis of serum cardiac injury markers (cTnT, cTnI, and CK-MB) revealed significant decreases in these indicators in the Nat10^-/-^ group (Fig. 7F). Hematoxylin-eosin (H&E) staining showed that pathological features such as cytoplasmic vacuolation, leukocytic infiltration, and interstitial edema were markedly reduced in the Nat10^-/-^ group (Fig. 7E). Given that uncontrolled inflammatory response is a major cause of heart damage, we employed various methods to assess the extent of inflammation. Immunohistochemical and immunofluorescence staining of myocardial tissue revealed a reduction in CD45^+^ immune cells and CD68^+^iNOS^+^ macrophages in the myocardium of the Nat10^-/-^ group (Fig. 7G–H). Flow cytometry analysis of myocardial tissue 12 h post-treatment showed reduced LPS-induced macrophage infiltration and a lower proportion of Ly6C^high^ macrophages in the Nat10^-/-^ group (Fig. 7I). Cytokine profiling of plasma from LPS-injected mice revealed a broad reduction in inflammatory cytokines, including IL-6, TNF-α, and IFN-γ in the Nat10^-/-^ group, confirmed by ELISA (Fig. 7J–K). Additionally, transcript levels of pro-inflammatory cytokines showed a smaller increase in the hearts of Nat10^-/-^ mice compared with Flox controls. (Fig. 7L)

Having demonstrated that NAT10 inhibition prevents cardiac dysfunction in an endotoxemia mouse model, we investigated the therapeutic potential of remodelin in endotoxemia treatment. Based on previous dosage reports [25] and LPS metabolic characteristics, remodelin treatment was initiated 3 days before LPS injection and continued throughout the 4-day follow-up period (Fig. S12A). As expected, remodelin consistently improved survival (Fig. S12B), enhanced cardiac function (Fig. S12C–F), and mitigated the inflammatory responses (Fig. S12G–I). Collectively, these results demonstrated that Nat10 knockdown or pharmacological inhibition markedly protects against LPS-induced heart injury in vivo.

## Discussion

Studies have highlighted the roles of NAT10 and ac4C modifications in various biological contexts, particularly in cancer and cardiovascular diseases [25, 27–29]. However, the functional significance of NAT10 in immune cells, such as macrophages, remains largely unknown. To address this, we used ac4C-seq, Ribo-seq, and RNA-seq to investigate the role of NAT10 in macrophages. We observed a significant increase in NAT10 protein levels in LPS-activated macrophages, whereas Nat10 mRNA levels remained unchanged, suggesting a post-transcriptional regulatory mechanism. We identified USP39 as a deubiquitinase of NAT10 that stabilizes its protein levels in activated macrophages. Furthermore, NAT10 was shown to catalyze ac4C modification of Ets2 mRNA at the 3′ UTR, enhancing its stability and translation efficiency. This mechanism is crucial for Ets2-dependent pro-inflammatory responses in macrophages, promoting their pro-inflammatory phenotype. Notably, in an ETM model, NAT10 inhibition reduces cytokine production, alleviates cardiac dysfunction, and improves the survival rate of LPS-challenged mice.

These findings are consistent with those of previous studies showing that ETS2 is crucial in macrophage-driven inflammation [26]. Our study is the first to directly link Ets2 mRNA stability and translation efficiency with Nat10-mediated ac4C modification, providing a novel layer of post-transcriptional regulation that complements previous work on the transcriptional roles of Ets2. Furthermore, the identification of USP39 as a regulator of NAT10 stability expands on the known mechanisms controlling NAT10 levels in macrophage. Mechanistically, we identified USP39 as the deubiquitinating enzyme responsible for stabilizing NAT10 through selective removal of K48-linked polyubiquitin chains, revealing a previously unrecognized regulatory axis in inflammation-induced post-translational control. These findings align with previous studies that reported the role of ac4C modification in regulating mRNA metabolism. However, we extend this knowledge to inflammatory responses in macrophages, highlighting the broader biological significance of ac4C.

Remodelin has been used in various studies as an inhibitor of NAT10’s lysine acetyltransferase activity [30, 31] and is considered a potential therapeutic agent for cancer treatment [32, 33]. Our findings suggest that remodelin is a promising agent for inhibiting macrophage overactivation and preventing inflammation-mediated organ damage. However, certain considerations must be addressed before its clinical application, including the determination of an appropriate therapeutic dosage and the identification and management of potential side effects.

Studies have demonstrated that RNA modifications, such as m6A, play critical roles in various biological processes in immune cells, including development, differentiation, activation, migration, and polarization [34]. However, studies on the role of the ac4C modification in immune cells or its involvement in related diseases remain limited. In light of the extensive research that has uncovered the critical role of ac4C modifications in cancer, cardiovascular diseases, and autoimmune disorders, further investigation of its function in immune cells is necessary. Identifying how ac4C influences immune cell behavior could open new avenues for therapeutic interventions aimed at modulating immune responses, improving immunological disease treatments, and enhancing the efficacy of immunotherapies.

This study provides insights into the role of Nat10 in pro-inflammatory macrophage activation via ac4C-mediated transcriptomic regulation. In the field of cardiovascular disease, NAT10 has been shown to be associated with myocardial remodeling and vascular remodeling [25, 35, 36], however, the function of NAT10 in inflammation-associated cardiac diseases remains poorly studied. Understanding the mechanisms by which NAT10 influences macrophage behavior can inform the development of novel therapeutic strategies aimed at controlling excessive inflammation in human cardiovascular diseases.

However, this study has some limitations. First, the specific mechanism underlying the LPS-mediated enhancement of USP39 and NAT10 interactions remains unclear. Additionally, the LysMCre-driven gene deletion affects both neutrophils and monocytes/macrophages. Studies have shown that neutrophils are associated with sepsis prognosis [37]. Currently, the role of Nat10 in neutrophil function remains poorly understood. Therefore, future studies are needed to determine its association with neutrophil function and its implications for ETM outcomes. While our current methods assess key inflammatory markers, we acknowledge broader septic inflammation dynamics may require advanced techniques like real-time leukocyte tracking in future studies. Furthermore, our results showed a moderate increase in Nat10 protein levels in IL-4-treated BMDMs, suggesting that Nat10 may influence anti-inflammatory macrophage polarization. However, this study exclusively focused on LPS-treated macrophages, necessitating further studies to explore the role of Nat10 in macrophage function across different activation states. While NAT10’s mechanistic role in TNF-α/IFN-γ-stimulated macrophage models remains underexplored, our preliminary findings demonstrate that genetic depletion of NAT10 significantly attenuates cytokine-driven inflammatory responses, suggesting broader regulatory functions beyond the currently characterized pathways.

In summary, our study is the first to reveal a crucial role for Nat10 in immune cell activation and inflammation-mediated cardiac injury.

It reveals a novel post-transcriptional mechanism of macrophage activation through NAT10-mediated ac4C modification of Ets2 mRNA. We demonstrated that NAT10 is stabilized by USP39, which enhances Ets2 mRNA stability and translation, driving macrophage pro-inflammatory responses. In an ETM model, NAT10 inhibition reduced inflammation and improved cardiac function, suggesting that NAT10 is a promising therapeutic target for controlling excessive inflammation. These findings provide new insights into the role of the ac4C modification in immune regulation and lay the foundation for future investigations into its broader implications in inflammatory diseases.

## Methods

### Animals

The C57BL/6 wild-type mice were sourced from SLAC Laboratory Animal (Shanghai, China), while the Nat10 ^flox/flox^ mice were generated by Cyagen (Suzhou, China) through the use of CRISPR/Cas9 technology. Additionally, LysM-Cre transgenic mice were obtained from the Jackson Laboratory. The conditional knockout mice were produced through the breeding of Nat10 ^flox/flox^ mice with LysM-Cre transgenic mice. All animals, aged between 6 and 8 weeks old and of the C57BL/6 background, were used in the study, regardless of sex. All procedures were conducted following the Guidelines for the Care and Use of Laboratory Animals by the Laboratory Animal Ethical Commission of Fudan University, and the study was approved by the Animal Ethical Committee of Zhongshan Hospital, Fudan University.

### Isolation and culture of BMDMs

The isolation and differentiation of mouse bone marrow-derived macrophages (BMDMs) were conducted in accordance with established protocols [38]. Bone marrow cells were extracted from mouse femurs using RPMI 1640 medium. The bone marrow cells were cultured in RPMI 1640 for a period of five hours, following which the non-adherent cells were transferred to a new dish containing complete RPMI 1640 medium (10% FBS, 1% penicillin/streptomycin) and 20 ng/ml M-CSF. The cells were then incubated for a period of seven days in order to facilitate differentiation into mature BMDMs.

### Cell lines

Wild-type RAW 264.7 and HEK293T cells were obtained from ATCC. The generation of RAW 264.7 cells with stable Nat10 knockdown, Nat10 overexpression, and Ets2 knockdown was achieved through the use of the CRISPR-Cas9 system, as developed by Cyagen (Suzhou, China). The guide sequences for Nat10 and Ets2 were identified as GCTCTTTTTTATAACACCAC-AGG and CCAGGCCATCAGGCCCTATG-AGG, respectively. The RAW 264.7 and HEK293T cells were cultivated in DMEM supplemented with 10% FBS and 1% penicillin-streptomycin, at 37 °C in a humidified incubator with 5% CO₂. The BMDMs and RAW 264.7 cells were treated with 100 ng/ml LPS (Invitrogen, Cat# TLRLEBLPS) or 100 ng/ml IL-4 (Sigma-Aldrich, Cat# SRP3211) for 24 h.

### Lentiviral, small interfering RNA (siRNA), and plasmid constructs

The Ets2 or shRNA plasmid-containing lentivirus (Santa Cruz Biotechnology, sc-37856-V and sc-76850-V) was purchased and used as reported previously for lentiviral transduction of bone marrow-derived macrophages (BMDMs) seeded in six-well plates at a density of 1 × 10⁶ cells per well following a five-day bone marrow cell culture. The BMDM culture medium was augmented with a lentiviral supernatant comprising either a control or mouse shEts2, in conjunction with 10 μg/mL polybrene (Millipore-Sigma), for a 12 h period. Thereafter, the medium was replaced. The transduced cells were harvested 48 h later.

siRNAs targeting ETS2 (CCTTCCAGACAACTATGAGAT), USP39 (CGCCAACTGTGAATTGCCTTT), and a non-specific control (NC) siRNA (UUCUCCGAACGUGUCACGUTT) were purchased from GenePharma (Shanghai, China). Plasmids encoding human or mouse Flag-NAT10, MYC-USP39, HA-Ub WT, and HA-Ub K48 mutant were obtained from ZORIN Biotech (Shanghai, China). The transient transfection of siRNA or plasmids into RAW264.7 or 293 T cells was conducted using Lipofectamine LTX or Lipofectamine 3000 Reagent (Thermo Scientific), in accordance with the instructions provided by the manufacturer.

### Co-immunoprecipitation (Co-IP)

The Pierce Crosslink Magnetic IP/Co-IP Kit (Thermo Scientific, USA, Cat# 88805) was employed in this study. An immunoprecipitation reaction was carried out utilizing 10 μg of either rabbit anti-mouse NAT10 antibody, USP39 antibody, or rabbit IgG antibody. The resulting immunoprecipitates, along with the input protein samples, underwent analysis through western blotting. The antibodies utilized in the co-immunoprecipitation reaction were anti-NAT10 antibody (ab194297) and anti-USP39 antibody (ab131244).

### Dot blot analysis of ac4C levels

The ac4C dot blot analysis was conducted in accordance with the methodology outlined by the original researchers [25]. RNA was denatured and spotted onto a nylon membrane (GE Healthcare, Cat# RPN203B, USA), then subjected to UV cross-linking at 150 mJ/cm² at 245 nm, repeated twice. Subsequently, the membrane was blocked at room temperature for 30 min with 5% nonfat dry milk, followed by incubation overnight at 4 °C with a 1:1000 dilution of anti-ac4C antibody (Abcam, Cat# ab252215, USA). Following three washes, the membrane was incubated with an HRP-conjugated anti-rabbit IgG secondary antibody, and levels of ac4C were detected via ECL. To serve as an internal standard, 0.02% methylene blue (Sigma-Aldrich, Cat# M4159) was applied to the membrane in 0.3 M sodium acetate at pH 5.2. The relative signal density was quantified using ImageJ software.

### Acetylated RNA immunoprecipitation sequencing (acRIP-seq)

The acRIP-seq procedure was conducted by Epibiotek (Guangzhou, China). The isolation of poly(A) RNA was performed using a two-step oligonucleotide selection method, and fragmentation was accomplished with the NEB-Next RNA fragmentation buffer, conducted at 94 °C for five minutes. The purified RNA, which was then held at 4 °C for a six-hour incubation period, was then incubated at the temperature with an anti-ac4C antibody in conjunction with Dynabeads Protein G (Abcam, USA). Following this, the RNA was subjected to an immunoprecipitation procedure in accordance with the specifications set out for acetylated RNA immunoprecipitation. Libraries were constructed using the NEBNext Ultra Directional RNA Library Prep Kit and sequenced on the Illumina NovaSeq platform (Illumina, CA, USA), following the manufacturer’s instructions. The distribution of signals (peak calling) was analyzed with MACS2, and the annotation of peaks and motifs was performed using DeepTools.

### Ribosome sequencing (Ribo-seq)

Ribosome profiling (ribo-seq) was performed by Epibiotek (Guangzhou, China). Administration of cycloheximide (100 μg/mL) was conducted on wild-type (WT) and Nat10 knockdown bone marrow-derived macrophages (BMDMs) for this study. The cells were lysed and treated with the Ribo-Seq Nuclease Mix in accordance with the instructions provided in the Epi™ Ribosome Profiling Kit protocol, as supplied by Epibiotek (product number R1814). To halt the reaction, SUPERase RNase Inhibitor (product number AM2696, supplied by Life Technologies) was added. Subsequently, the harvested RNA was subjected to depletion of rRNA through utilization of the Ribo-RNA Depletion Kit (Epibiotek, Cat# R1805), in accordance with the instructions set forth by the manufacturer. Following this, RNA was extracted from the residual material through the application of the RNA Clean & Concentrator-5 Kit (ZYMO, Cat# R1016). Thereafter, the purified RNA was prepared for library construction and quality control through the utilization of the Bioptic Qsep100 Analyzer (Bioptic Inc.).

### Polysome profiling

The polysome profiling procedure was conducted in adherence to the methodology outlined in BioProtocol (www.bio-protocol.org/e2126). In summary, ice-cold 1× PBS was utilized to rinse NAT10 control or depleted BMDMs (6 × 100 mm dishes). Upon the removal of residual PBS, the cells were incubated in 2 mL PBS containing 100 μg/ml cycloheximide (CHX, Selleck, TX, USA) for 10 min at 37 °C. The cell extract, comprising 1 ml of cytoplasm, was subjected to an 11 ml sucrose gradient comprising concentrations ranging from 10% to 50%. The gradient was centrifuged for 90 min at 39,000 rpm, 4 °C, using a Beckman SW-41Ti rotor. The fractions were then evaluated using a Brandel apparatus (Florida, USA) to ascertain their absorbance at 254 nm. The transcriptional activity of Ets2 was evaluated using reverse transcription-polymerase chain reaction (RT-PCR) on the collected fractions.

### mRNA stability assay

To assess mRNA stability, cells were treated with 5 μg/ml actinomycin D (ActD, Catalog #A9415, Sigma, US), followed by RNA isolation for RT-qPCR analysis. The resulting data were then employed to determine the half-life (t1/2) of Ets2 mRNA.

### Protein decay assay

Before incubation in a cycloheximide-containing medium, macrophages were treated with either PBS or lipopolysaccharide (LPS) at a concentration of 100 ng/ml, which is the standard dose used in vitro for studies of this nature. The incubation period proceeded for the indicated time, during which the cycloheximide-containing medium was maintained. To facilitate protein extraction, the cells were lysed in RIPA buffer following the incubation period. Subsequently, Western blotting was employed to assess protein expression levels, with band intensities quantified by ImageJ software to ensure accuracy in densitometric analysis and data presentation.

### RNA pull-down

Biotinylated RNA fragments containing Ets2 sequences with predicted Nat10 binding sites were synthesized on a commercial scale. The Pierce Magnetic RNA-Protein Pulldown Kit (Thermo Fisher Scientific, Cat# 20164, USA) was employed according to the manufacturer’s instructions. Biotin-labeled RNA hybrids were bound to streptavidin magnetic beads and incubated with cell lysates in a binding buffer (20 mM Tris-HCl, pH 7.5; 50 mM NaCl; 2 mM MgCl₂). The incubation period was followed by a washing step with a buffer (20 mM Tris-HCl, pH 7.5; 10 mM NaCl; 0.1% Tween-20) and the elution of the RNA-protein complexes with SDS-loading buffer. Subsequently, Western blot analysis was performed to assess NAT10 expression in the binding supernatants and eluates. The biotinylated RNA oligo sequences are detailed in the supplementary table (S1).

### Flow cytometry assay

The cells were derived from lipopolysaccharide (LPS)–stimulated BMDMs and RAW 264.7 cell lines. Prior to initiating subsequent assays, an anti-mouse CD16/32 antibody was employed to block Fc receptors, thereby ensuring that the assays were conducted with the requisite specificity. Subsequently, the cells were incubated with PE-conjugated CD80 and FITC-conjugated CD86 antibodies, after which the activation markers were evaluated. For cells isolated from mouse hearts, a single-cell suspension was prepared by enzymatic digestion and dissociation of heart tissue, by the established protocols. Subsequently, the cells were counted and stained with a panel of antibodies from BioLegend (San Diego, CA, USA), including Pacific, Blue-conjugated anti-mouse CD45, APC/cyanine7-conjugated anti-mouse CD11b, PE-conjugated anti-mouse F4/80, and FITC-conjugated anti-mouse LY6C. To ensure comprehensive and accurate cell phenotyping, the cell populations were analyzed using a BD FACSAria III flow cytometer, and the data were processed with FlowJo V10 software (TreeStar, Ashland, OR, USA).

### Confocal microscopy

The BMDMs were seeded onto glass slides and subsequently washed with phosphate-buffered saline (PBS). After a period of 15 min, the samples were fixed in a solution of 4% paraformaldehyde (PFA). Following fixation, a process of permeabilization was initiated using a solution of PBS and Triton X-100 (0.1%), which was continued for a period of 15 min. Subsequently, a blocking solution comprising 1 mg/ml BSA in PBS was introduced for 30 min at room temperature. Primary antibodies were applied for 24 h at 4 °C, followed by a one-hour incubation of secondary antibodies at room temperature. Cell nuclei were stained with 4’,6-diamidino-2-phenylindole (DAPI) to facilitate visualization. Fluorescence images were captured with a confocal laser scanning microscope (Olympus, Japan).

### Western blot analysis

In line with previous research, the following antibodies were utilized: anti-NAT10 (Abcam, ab194297), anti-ETS2 (Abcam, ab219948), anti-FLAG (Sigma, F1804), anti-Myc-tag (Abcam, ab32), anti-HA (Abcam, ab314237), anti-iNOS (Abcam, ab178945), anti-CD206 (Abcam, ab64693), anti-USP39 (Abcam, ab131244), anti-Histone H3 (Abcam, ab64693), anti-ubiquitin (Proteintech, Cat# 10201-2-AP), anti-GAPDH (Proteintech, Cat# 60004-1-Ig), and anti-beta Actin (Proteintech, Cat# 60004-1-Ig).

### RNA extraction followed by real-time qPCR

Total RNA was extracted from freshly harvested tissues or cells using the TRIzol reagent (Invitrogen, Cat# 15596026), and cDNA was synthesized using the TOYOBO qPCR reverse transcription kit (Cat# FSQ-101), and the TOYOBO real-time PCR kit (Cat# QPK-201) with the Roche real-time PCR system. The resulting cDNA was subjected to real-time PCR analysis with gene-specific primers and the SYBR Green master mix kit. Gene expression data were normalized using β-actin or GAPDH RNA as internal controls. Primer sequences are listed in the Supplementary Table S1.

### Cytokine measurement

Concentrations of TNF-α, IL-6, IL-1β, and IFN-γ in cell culture media and mouse serum were quantified using ELISA kits, following the manufacturer’s standard protocols.

### Lipopolysaccharide (LPS)-induced endotoxemia

The mice were administered either phosphate-buffered saline (PBS) or lipopolysaccharide (LPS, 20 mg/kg; E. coli, O111, Sigma-Aldrich, #L3024) by intraperitoneal injection, and the subsequent progress was observed for a period of up to 96 h. Cardiac function was evaluated in vivo via echocardiography 12 h post-injection. Blood samples were obtained via orbital puncture to conduct ELISA and other biochemical analyses. The hearts were then excised for pathological examination and additional assays.

### Cardiac function assessment

12 h following the administration of the LPS treatment, the mice were anesthetized to facilitate an accurate assessment of their cardiac function. The Vevo 770 ultrasound system (VisualSonics, Toronto, Canada) was utilized to measure heart rate (HR), left ventricular ejection fraction (LVEF), left ventricular fractional shortening, left ventricular cardiac output (LVCO), and left ventricular stroke volume (LVSV).

### Murine plasma analysis

Plasma samples were obtained 12 hours following the administration of lipopolysaccharide (LPS) treatment. Plasma cytokine levels were quantified using RayBio Mouse Cytokine Antibody Array kits (RayBiotech, Norcross, GA, USA). The data were analyzed by GeneTools software (Syngene).To ensure comparability, the data were normalized using Z-score standardization. A heatmap illustrating the standardized levels of inflammatory cytokines was constructed using GraphPad Prism 9.

### Histology, immunohistochemical and immunofluorescence analysis

The hearts were fixed in 4% paraformaldehyde, sectioned, and stained with hematoxylin and eosin (HE) to conduct a tissue morphology analysis. For immunohistochemical analysis, the sections were incubated with an anti-CD45 antibody (ProteinTech, Cat. No. 28463-1-AP) at 4 °C for 15 . Subsequently, the sections were treated with horseradish peroxidase (HRP)-conjugated anti-rabbit IgG secondary antibody and visualized using 3,3’-diaminobenzidine (DAB). The presence of inflammatory cells was identified using antibodies against CD68 (ab283654, Abcam) and iNOS (ab178945, Abcam), by the instructions provided by the manufacturer. Subsequently, cell nuclei were counterstained with 4’,6-diamidino-2-phenylindole (DAPI; C1006, Beyotime). Fluorescence images were acquired with an Olympus fluorescence microscope (Japan).

### Statistical analysis

An unpaired Student’s *t*-test was employed to ascertain the statistical significance of mean differences between groups. In the case of multi-group analyses, a one-way or two-way ANOVA was employed, followed by Bonferroni’s post hoc test to control for type I error. The statistical analyses were conducted using GraphPad Prism (GraphPad Prism 9 Software Inc., San Diego, CA), which is recognized for its robust capabilities. In the case of the Kaplan-Meier analysis, the *P* values were evaluated using the log-rank test for Meier curves. The following notation is used in the figures: *P < 0.05, **P < 0.01, ***P < 0.001 ***, and *P* < 0.0001.

## Supplementary information

Supplementary Figures and Tables

Original Western blots

## Data Availability

All data needed to evaluate the conclusions in the paper are present in the paper and/or the Supplementary Materials. The sequencing raw data generated in this study have been deposited in the Sequence Read Archive (SRA) at the NCBI Center with the accession numbers PRJNA1162481, PRJNA1162768, and PRJNA1163436.
