# Supplementary material for: Impact of N-acetyltransferase 10 on macrophage activation and inflammation-induced cardiac dysfunction
Source: Cell Death Dis. 2025 Jul 1;16(1):471. doi: 10.1038/s41419-025-07796-6 (PMC12216202; doi:10.1038/s41419-025-07796-6)
Supplement: Supplementary file 1 — Supplementary Figures and Tables [file 41419_2025_7796_MOESM1_ESM.docx]

**Supplementary Materials for**

**Impact of N-Acetyltransferase 10 on Macrophage Activation and Inflammation-Induced Cardiac Dysfunction**

Zilong Xiao et al.

Corresponding author: Yixiu Liang yixiu.liang@imperial.ac.uk; Yangang Su su.yangang@zs-hospital.sh.cn

**This PDF file includes:**

Supplementary Text

Tables S1 #

Figs. S1 to S12#

# **sTABEL1 Sequences of Primers used in the study**

| RT-qPCR | | | |
| --- | --- | --- | --- |
| Gene | Species | FW | RW |
| Nat10 | Mouse | GCGGCAGAGGTCTCTTTTTGT | GTGACTGCTAAATCCCAGCTC |
| Ets2 | Mouse | CCTGTCGCCAACAGTTTTCG | TGGAGTGTCTGATCTTCACTGA |
| Nos2 | Mouse | GTTCTCAGCCCAACAATACAAGA | GTGGACGGGTCGATGTCAC |
| Tnf-α | Mouse | TCAGCCTCTTCTCATTCCTG | CAGGCTTGTCACTCGAATTT |
| IL-6 | Mouse | CCTCTCTGCAAGAGACTTCCA | AGAATTGCCATTGCACAACTCT |
| IL-1β | Mouse | CCAAAAGATGAAGGGCTGCT | ACAGAGGATGGGCTCTTCTT |
| Ifn-**γ** | Mouse | GCGTCATTGAATCACACCTG | TGAGCTCATTGAATGCTTGG |
| β-actin | Mouse | CCGTGAAAAGATGACCCAGA | TACGACCAGAGGCATACAG |
| acRIP-qPCR | | | |
| Ets2 | Mouse | GTCAGTTTATGTACAGTGGAGCC | AAACCGTCATGTTGGCTGAC |
| NAT10-RIP-qPCR | | | |
| Ets2 | Mouse | GAGTGGACACCACTGAGGC | GGCGACAGGGGCCACT |

# **Supplementary Figures**


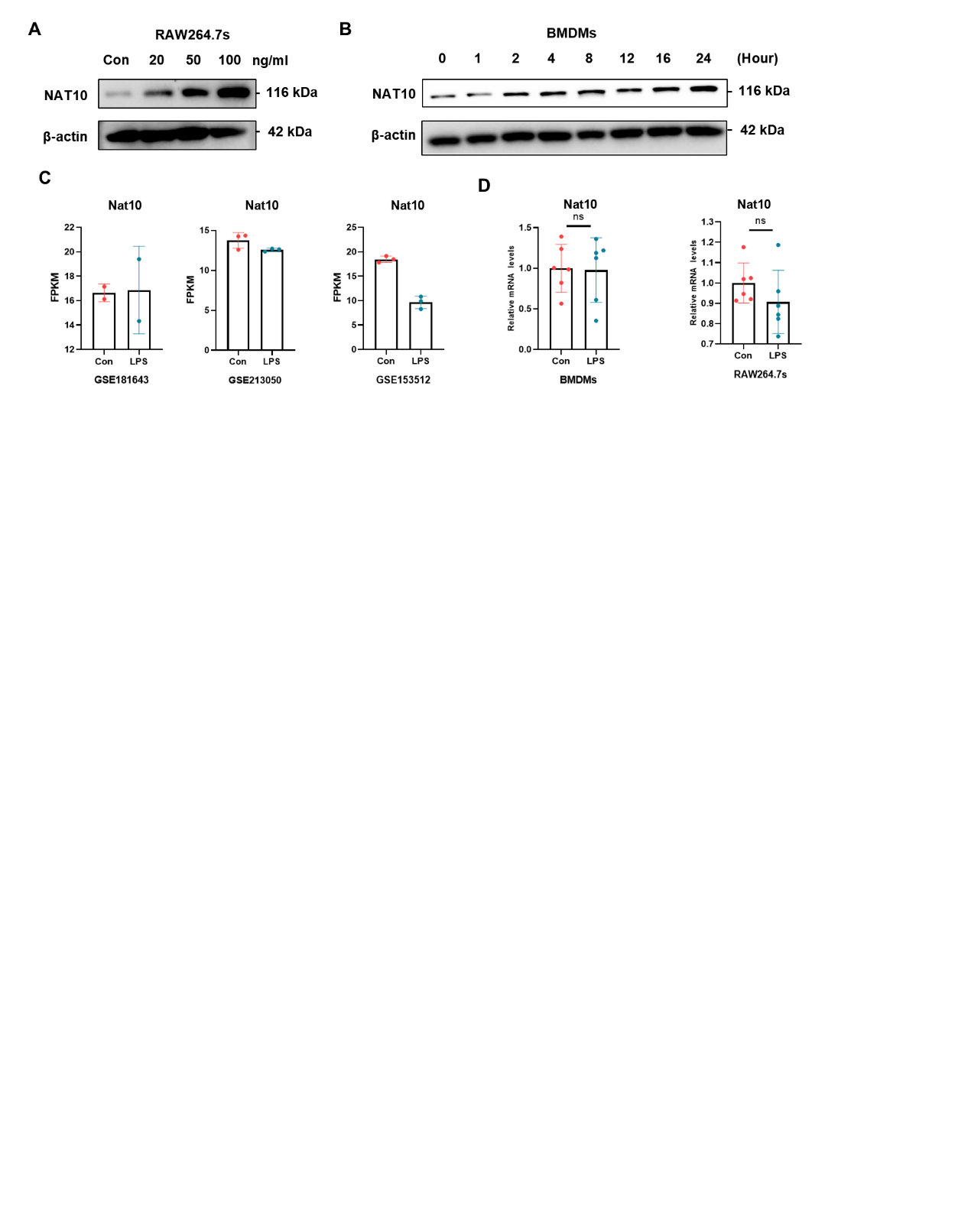


**Figure S1**: Changes in Nat10 RNA and protein levels in macrophages are not consistent following LPS stimulation.
**(A)** Western blot analysis of NAT10 protein in RAW264.7 cells treated with different doses of LPS.
**(B)** Western blot analysis of NAT10 protein levels in BMDMs at different time points following LPS treatment.
**(C)** Changes in Nat10 RNA levels in BMDMs following LPS treatment as observed in three GEO datasets.
**(D)** RT-qPCR analysis of Nat10 RNA levels in BMDMs and RAW264.7 cells after 24 hours of treatment with LPS (n=6 per group).

Data are shown as mean ± SD. Statistical analyses were performed using the t-test.


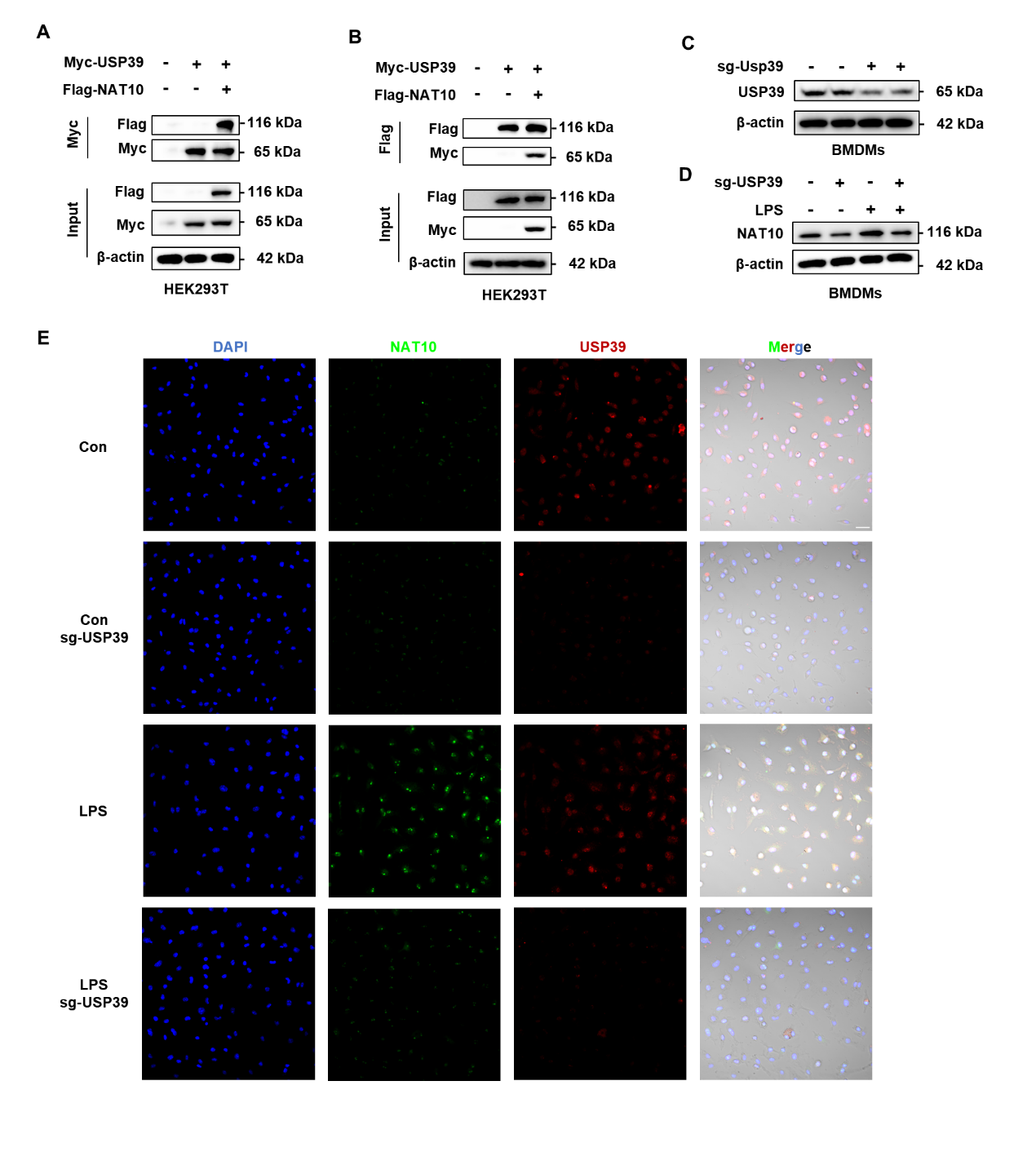
**Figure S2: USP39 interacts with NAT10 and affects its degradation.**
**(A, B)** Co-immunoprecipitation (Co-IP) analysis shows the association of USP39 with NAT10 in 293T cells transfected with Myc-tagged USP39 and Flag-tagged NAT10.
**(C)** Validation of USP39 knockdown efficiency in BMDMs following lentiviral transduction.
**(D, E)** Western blot and immunofluorescence analyses demonstrate that knockdown of USP39 reduces the LPS-induced increase in NAT10 protein levels.(scalebar=10um)

**
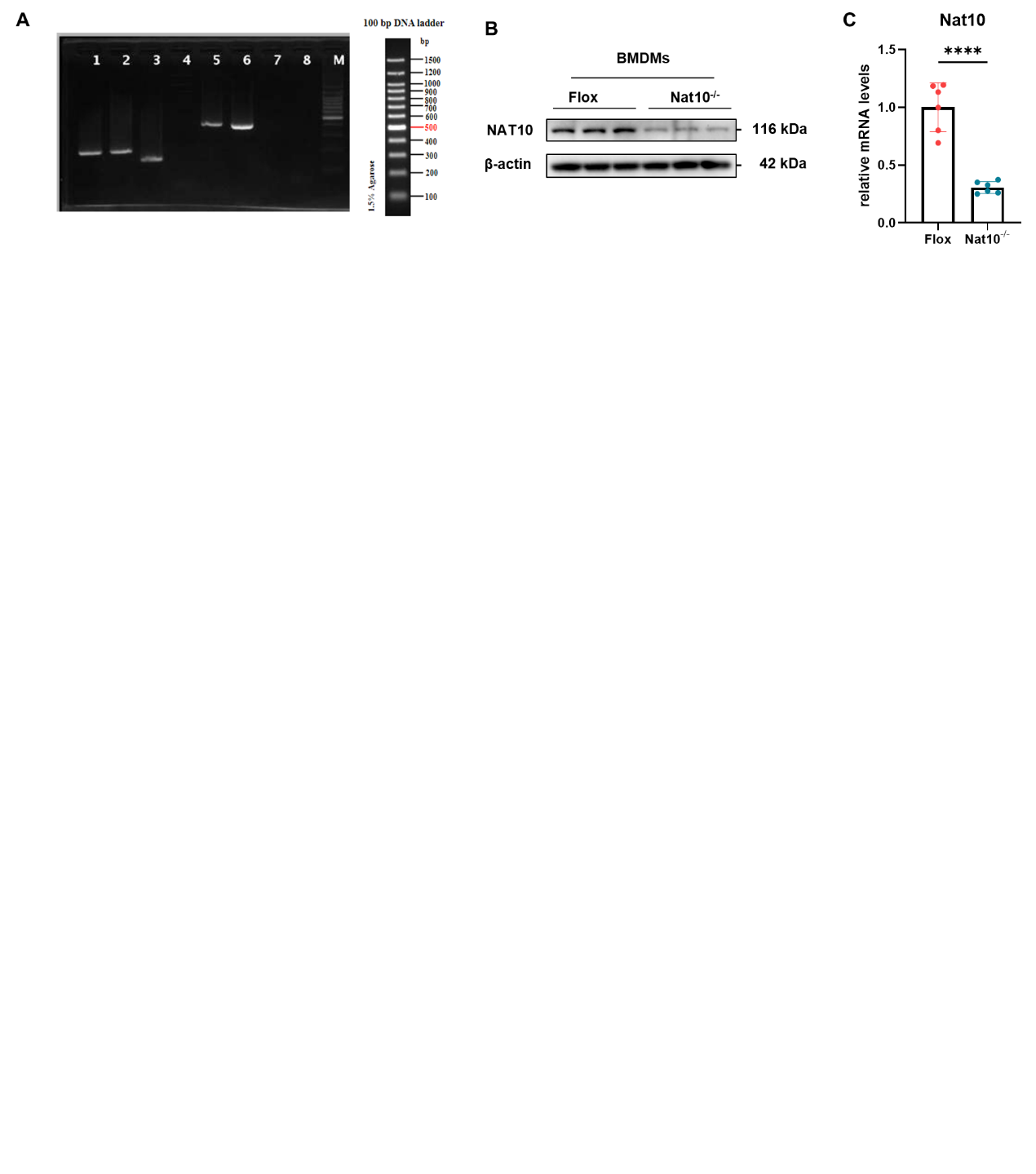
Figure S3: Generation and validation of Nat10-/- mice.
(A)** Confirmation of Nat10 deficiency by assessing genomic DNA. Lane 1/5 was Flox control, lane 2/6 was Cre-Flox, lane 3/7 was WT, and lane 4/8 was water. In lanes 1-4, Nat10-F2 and Nat10-R2 were used as primers. Lane 5-8 used Nat10-Cre-F and Nat10-Cre-R as primers.
**(B-C)** Nat10 knockout confirmed by RT-qPCR and Western blot analysis. Data are shown as mean ± SD. Statistical analyses were performed using the t-test.

#
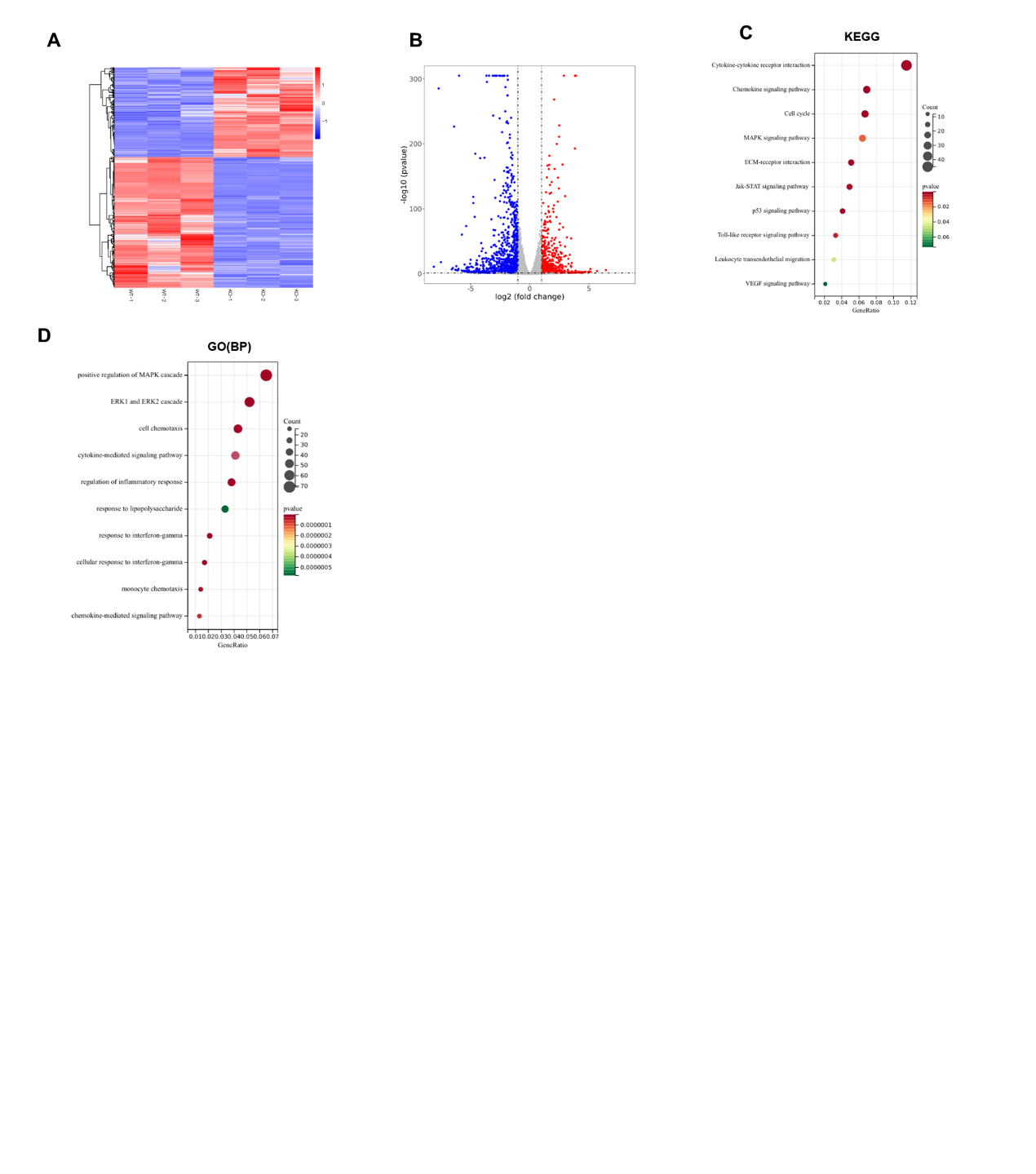


**Figure S4 Gene Expression Changes in the Nat10-/- BMDMs.**

**(A)** Heatmap of differentially expressed transcripts in LPS-treated Flox and Nat10-/- BMDMs.

**(B)** Volcano plot showing changes in mRNA expression after Nat10 knockout in LPS-treated BMDMs.

**(C)** Top-ranked KEGG pathways of downregulated gene sets are presented. **(D)** Top-ranked GO biological process enrichment analysis of downregulated gene sets are presented.


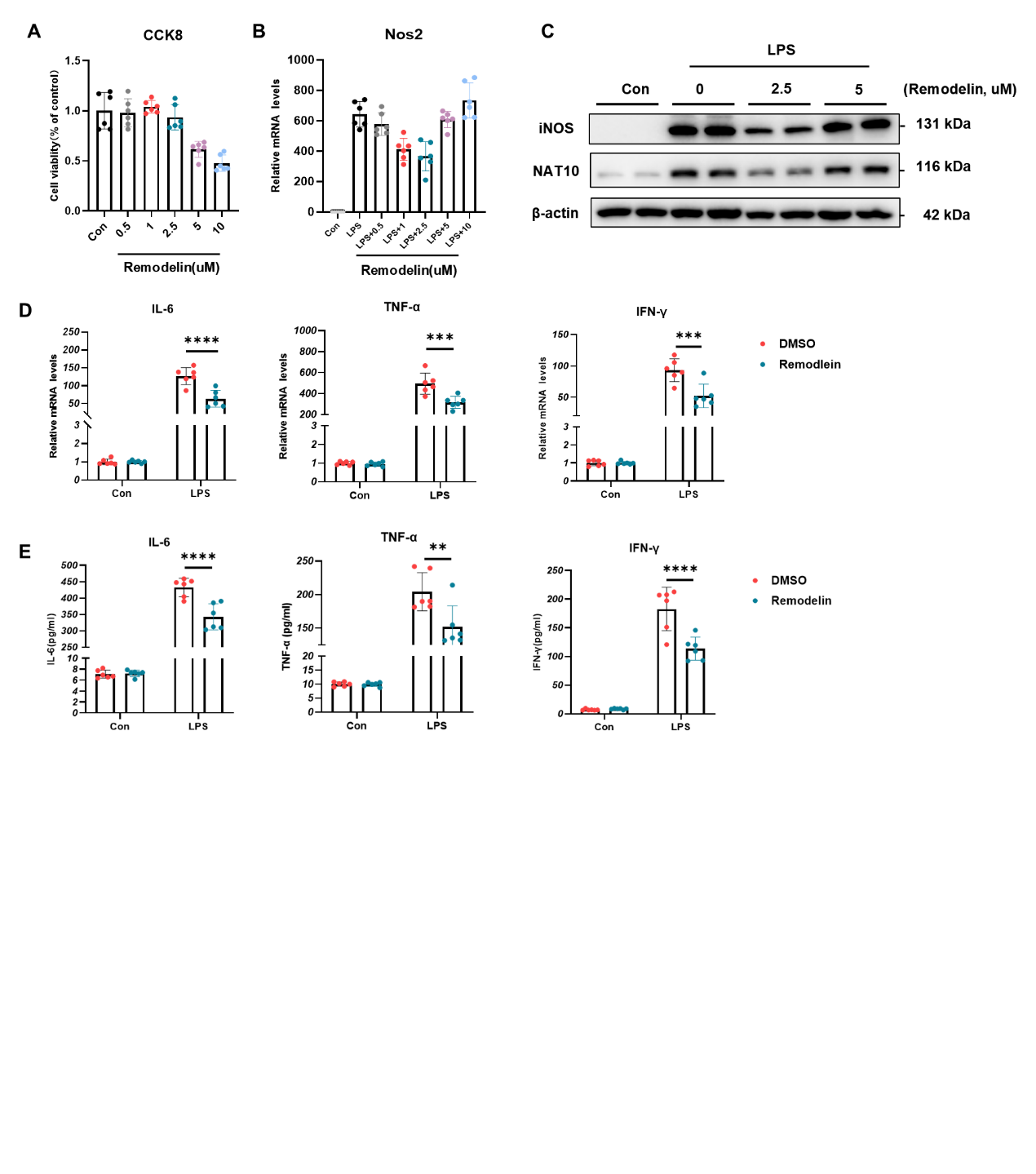
**Figure S5: Remodelin inhibits LPS-induced inflammation in BMDM cells.
(A)** CCK8 assay analysis of the cytotoxicity of Remodelin in BMDMs (n = 6 per group).
**(B)** RT-PCR analysis of Nos2 expression in BMDMs pretreated with Remodelin for 2 hours, followed by treatment with LPS for 24 hours(n = 6 per group).
**(C)** Immunoblot analysis of INOS and NAT10 in BMDMs.
**(D)** RT-PCR analysis showing mRNA expression of cytokines in BMDMs pretreated with 2.5 μM Remodelin for 2 hours, followed by treatment with LPS for 24 hours(n = 6 per group).**(E)** ELISA analysis of cytokine secretion in the culture medium of BMDMs(n = 6 per group).

Data are shown as mean ± SD. Statistical analyses were performed using the One-way two-sided ANOVA.

**
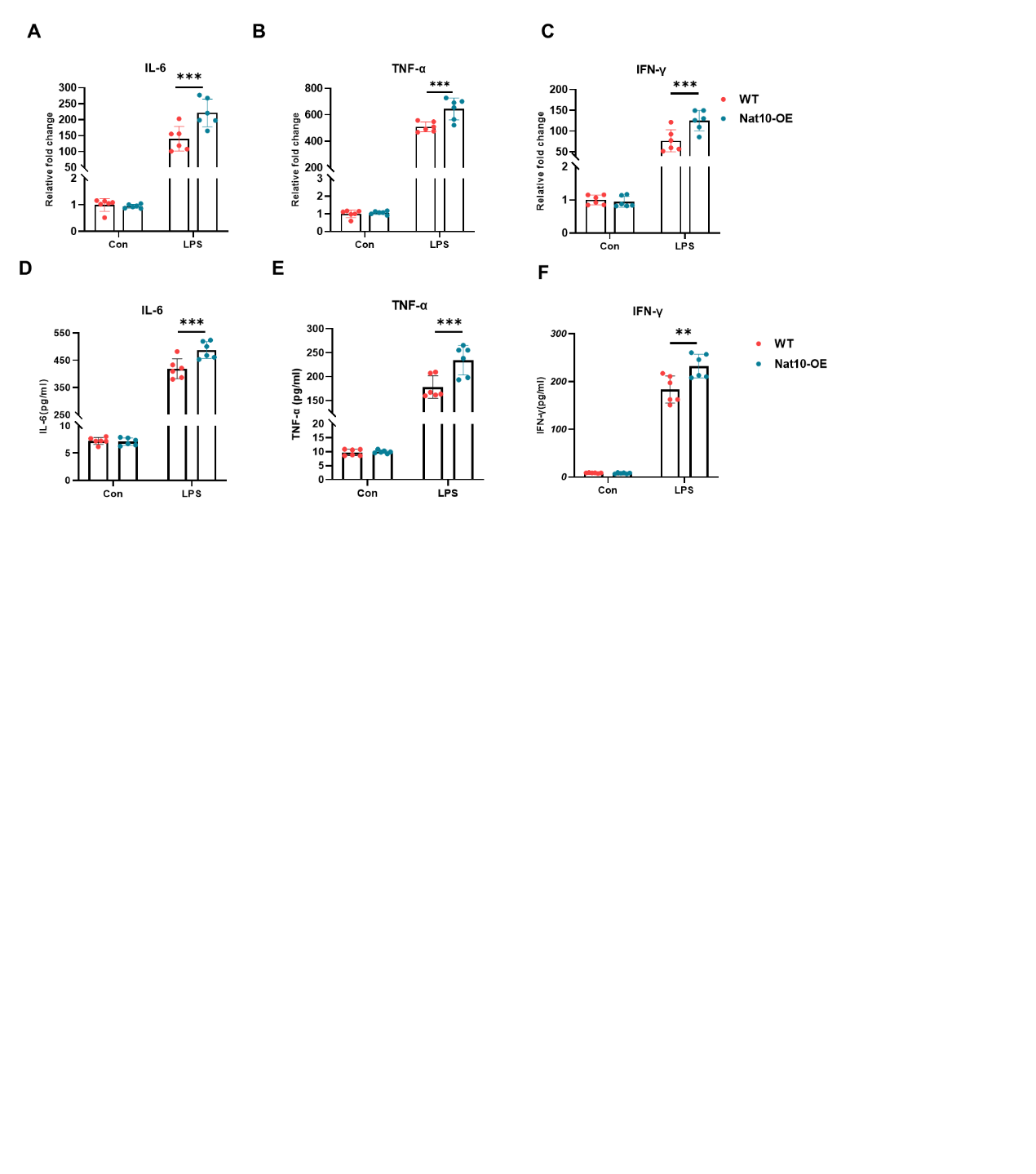
Figure S6**: Nat10 overexpression induced exacerbation of macrophage activation in RAW264.7 cells.
**(A-C)** Real-time PCR analysis of mRNA expression of cytokines in WT or Nat10-stable overexpressing RAW264.7 cells with or without LPS treatment (n = 6 per group).
**(D-F)** ELISA analysis of cytokine secretion in WT or Nat10-stable overexpressing RAW264.7 cells with or without LPS treatment (n = 6 per group).
Data are presented as mean ± SD. Statistical analyses were performed using two-way ANOVA (two-sided).

**
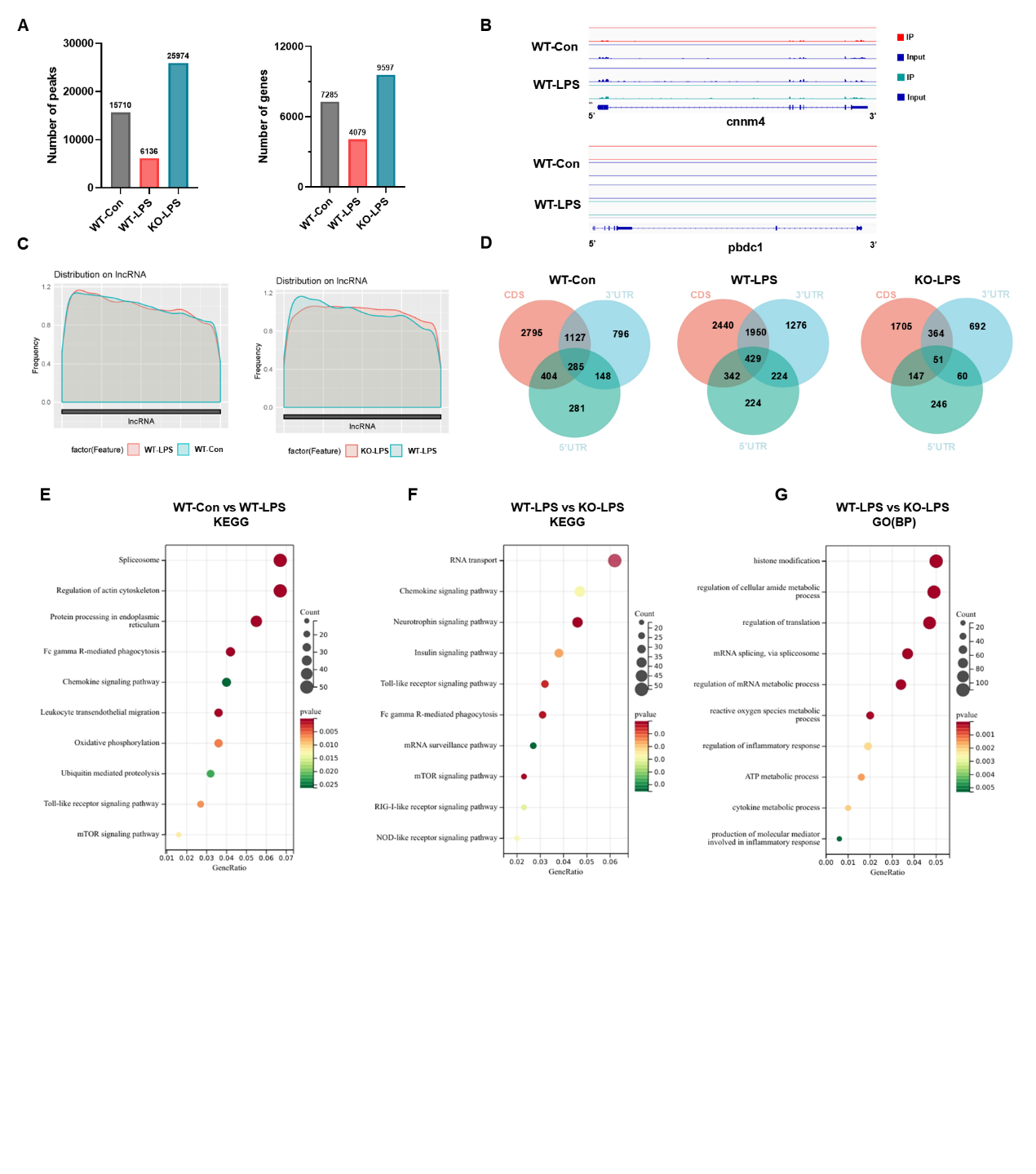
Figure S7: Further analysis of acRIP-seq.
(A)** Number of ac4C peaks and ac4C-modified genes identified in acRIP-seq in WT-CON , WT-LPS, and KO-LPS groups.
**(B)** Integrative Genomics Viewer (IGV) tracks displaying ac4C-seq results for Cnnm4 and Pbdc1 mRNA. **(C)** Distribution of acetylated positions in lncRNA between WT-LPS vs. WT-CON and KO-LPS vs. WT-LPS groups.
**(D)** Venn diagram showing acetylation marks across transcript regions in WT-LPS vs. WT-CON and KO-LPS groups.
**(E)** KEGG enrichment analysis for hyperacetylated and hypoacetylated transcripts between WT-LPS and WT-CON groups.
**(F)** KEGG enrichment analysis for hyperacetylated and hypoacetylated transcripts between KO-LPS and WT-LPS groups.
**(G)** GO biological process analysis for hyperacetylated and hypoacetylated transcripts between KO-LPS and WT-LPS groups.


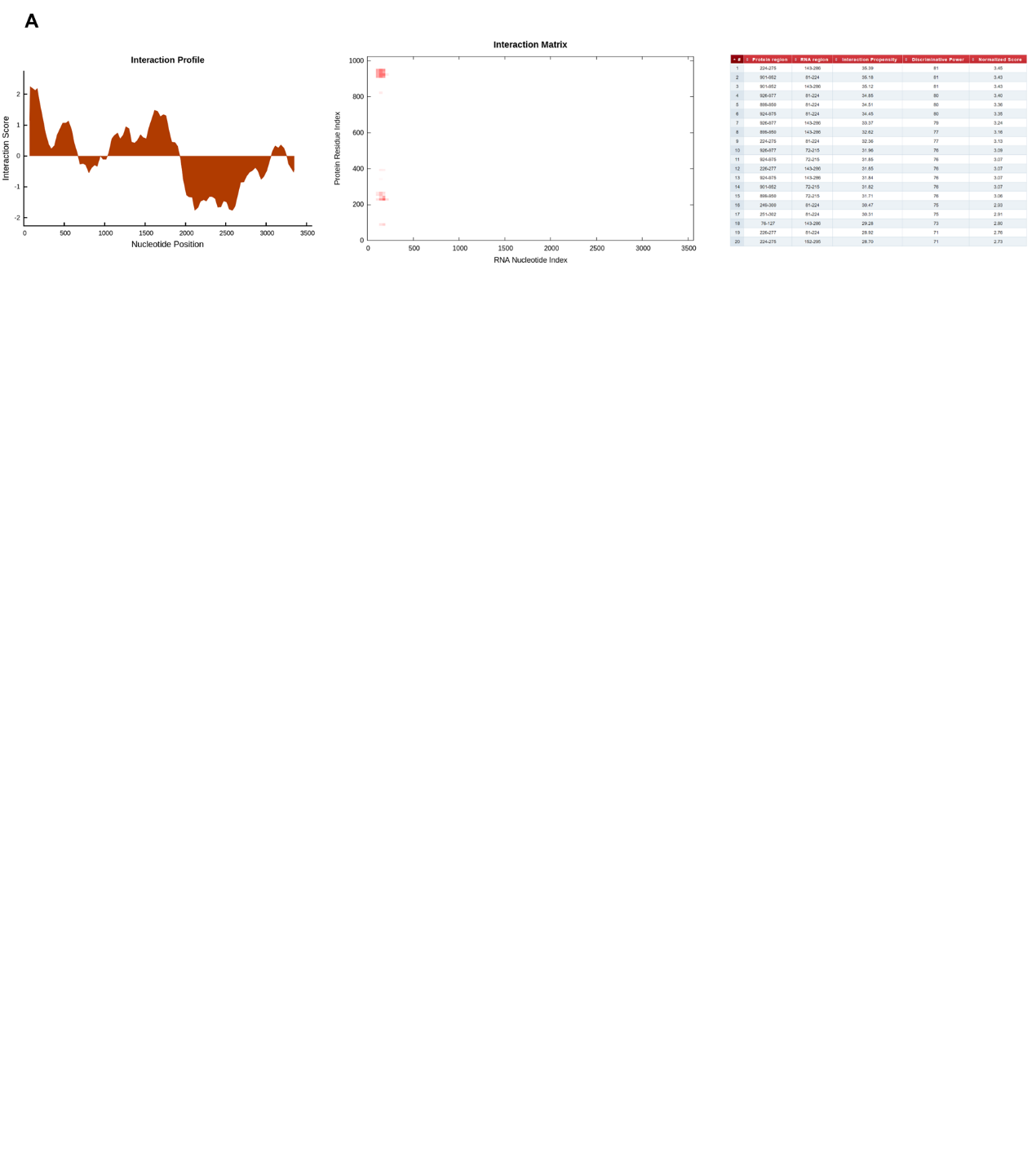


**Figure S8** The prediction of the binding sites between NAT10 and Ets2 using the catRAPID algorithm


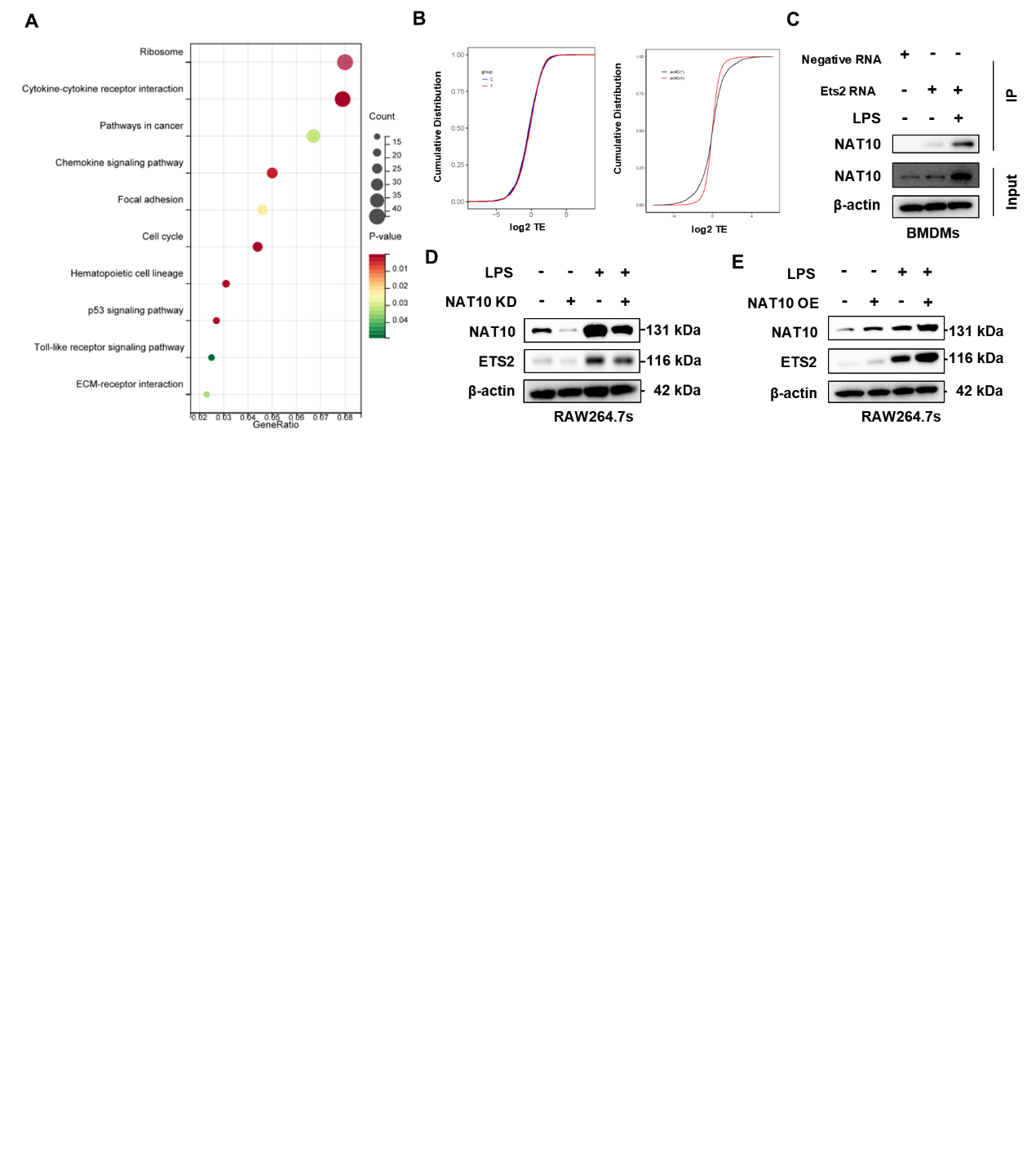
**Figure S9**: **NAT10-mediated ac4C RNA acetylation enhances Ets2 mRNA stability and translation efficiency.
(A)** GO biological process analysis of differentially expressed genes (DEGs) in Ribo-seq.
**(B)** Cumulative distribution function (CDF) plot of mRNA-normalized ribosome footprint reads (translation efficiency, T.E.) for ac4C(-) transcripts in Nat10^-/-^vs. Flox BMDMs (left); and ac4C(-) and ac4C(+) transcripts in Nat10^-/-^ vs. Flox BMDMs (right).
**(C)** RNA pull-down assay evaluating the interaction between Nat10 and Ets2 in BMDMs.
**(D)** Western blot analysis of Ets2 in Nat10 knockdown RAW264.7 cells.
**(E)** Western blot analysis of Ets2 in Nat10-overexpressing RAW264.7 cells.

**
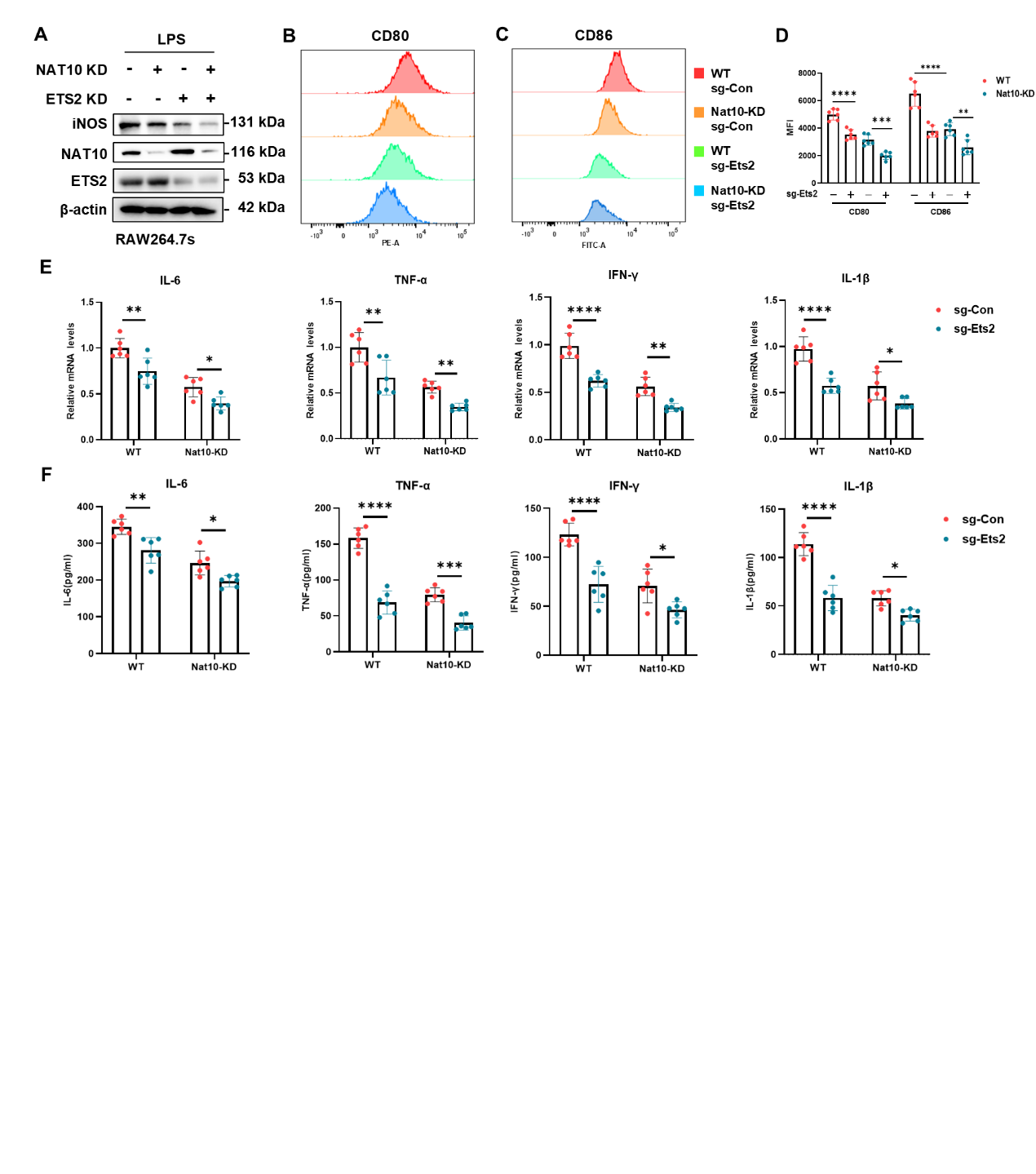
Figure S10 The Nat10/Ets2 axis is involved in the mechanism of LPS-induced macrophage activation.**(A) Western blot detection of iNOS, NAT10, and ETS2 protein levels in RAW264.7 cells. WT and Nat10 stable knockdown RAW264.7 cells were transfected with or without Ets2 lentivirus.
(B-D) Flow cytometry analysis of macrophage surface markers CD80 and CD86 expression in RAW264.7 cells of each group(n=6 per group).
(E) Real-time PCR analysis of mRNA expression of cytokines in RAW264.7 cells of each group(n=6 per group).
(F) ELISA analysis of cytokine secretion in the culture medium of RAW264.7 cells of each group(n=6 per group).
Data are shown as mean ± SD. Statistical analyses were performed using two-way two-sided ANOVA.


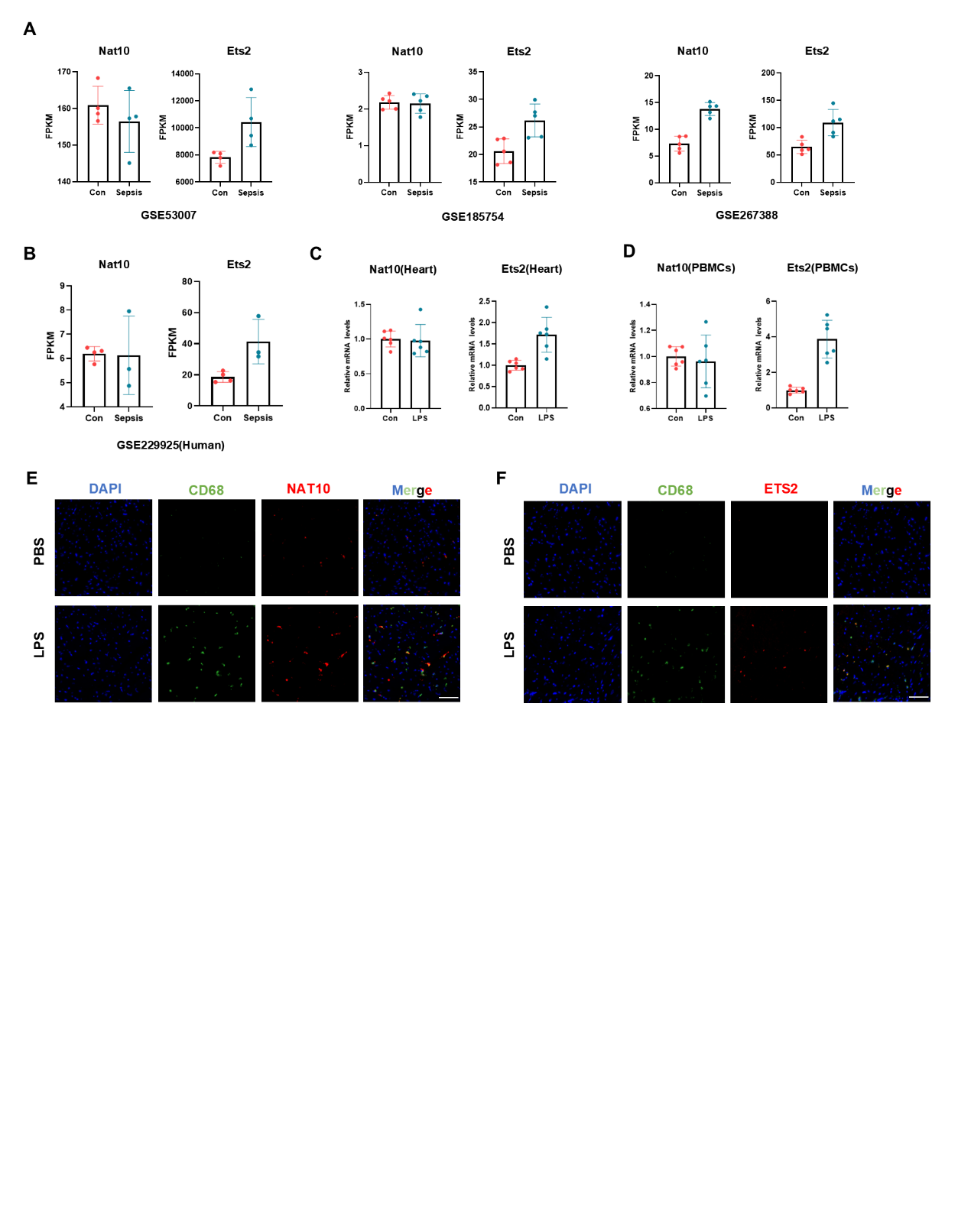
**Figure S11**: **Expression of Nat10 and Ets2 in myocardial tissue and PBMCs during endotoxemia.
(A)** Changes in Nat10 and Ets2 RNA expression in myocardial tissue of septic mice from GEO datasets.
**(B)** Changes in NAT10 and Ets2 RNA expression levels in myocardial tissue from septic patients with reduced left ventricular ejection fraction from GEO datasets.
**(C)** qPCR analysis of changes in Nat10 and Ets2 RNA expression in mouse myocardial tissue 12 hours after LPS treatment.
**(D)** qPCR analysis of changes in Nat10 and Ets2 RNA expression in mouse PBMCs 12 hours after LPS treatment.
**(E)** Immunofluorescence staining of CD68 (green) and NAT10 (red) in the hearts of PBS- or LPS-injected mice. (scale bar = 100um).
**(F)** Immunofluorescence staining of CD68 (green) and ETS2 (red) in the hearts of PBS- or LPS-injected mice. (scale bar = 100um).

**
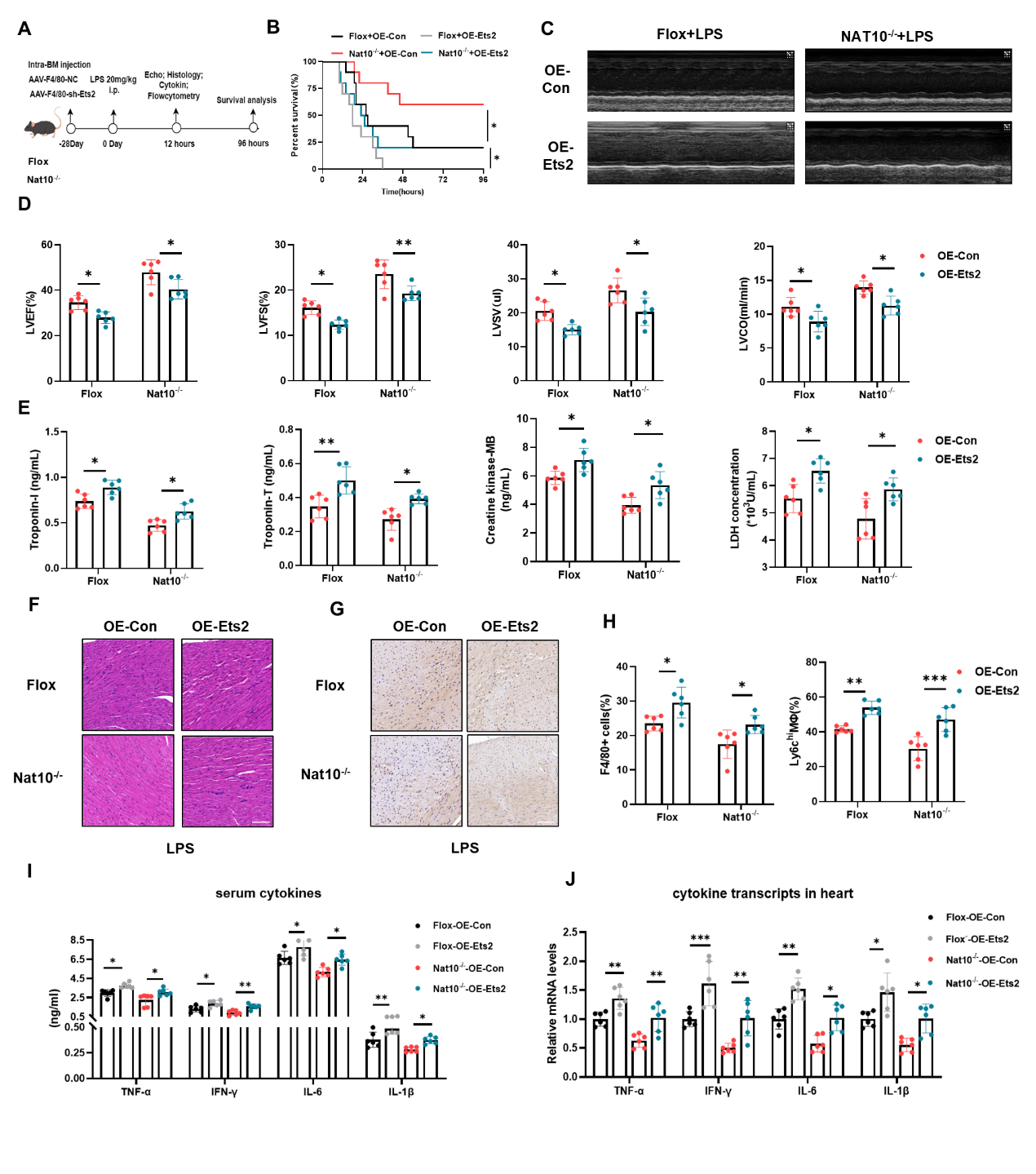
Figure S12**: **Remodelin protects the heart during endotoxemia.
(A)** Experimental design: C57BL/6 mice were randomly divided into four groups: (i) untreated mice receiving 0.1% DMSO vehicle control (PBS; n = 10); (ii) untreated mice treated with intraperitoneal injection of 20 mg/kg/day Remodelin; (iii) LPS-challenged mice receiving 0.1% DMSO vehicle control (n = 10); (iv) LPS-challenged mice treated with intraperitoneal injection of 20 mg/kg/day Remodelin (n = 10).
**(B)** Kaplan-Meier survival curve analysis of mice treated with Remodelin following LPS intervention (n = 10 male mice per group).
**(C, D)** Representative echocardiographic images and statistical analysis of Left Ventricular Ejection Fraction, Fractional Shortening, Stroke Volume and Cardiac Output in LPS-challenged mice treated with or without Remodelin (n = 6 male mice per group).
**(E)** Serum levels of cardiac troponin-T, troponin-I, creatine kinase-MB and lactate dehydrogenase (n = 6 male mice per group).
**(F)** Representative photomicrographs of ventricular tissues stained with hematoxylin and eosin (H&E). (scale bar = 100um)
**(G)** Representative immunohistochemical staining of CD45 in left ventricular myocardium. (scale bar = 100um)
**(H)** Quantification showing the ratio of CD11B^+^ F4/80^+^ macrophages and F4/80^+^ Ly6C^high^ macrophages in each group (n = 6 male mice per group).
**(I)** ELISA analysis of cytokine levels in serum. (n = 6 male mice per group).
**(J)** Real-time PCR analysis of cytokine expression in heart tissue (n = 6 per group).

Data are shown as mean ± SD. Statistical analyses were performed using the Log-rank test (B), student t-test (H), and two-way two-sided ANOVA (D, E, I, J).
