# Supplementary material for: Impact of N-acetyltransferase 10 on macrophage activation and inflammation-induced cardiac dysfunction
Source: Cell Death Dis. 2025 Jul 1;16(1):471. doi: 10.1038/s41419-025-07796-6 (PMC12216202; doi:10.1038/s41419-025-07796-6)
Supplement: Supplementary file 2 — Original Western blots [file 41419_2025_7796_MOESM2_ESM.pdf]

Figure1

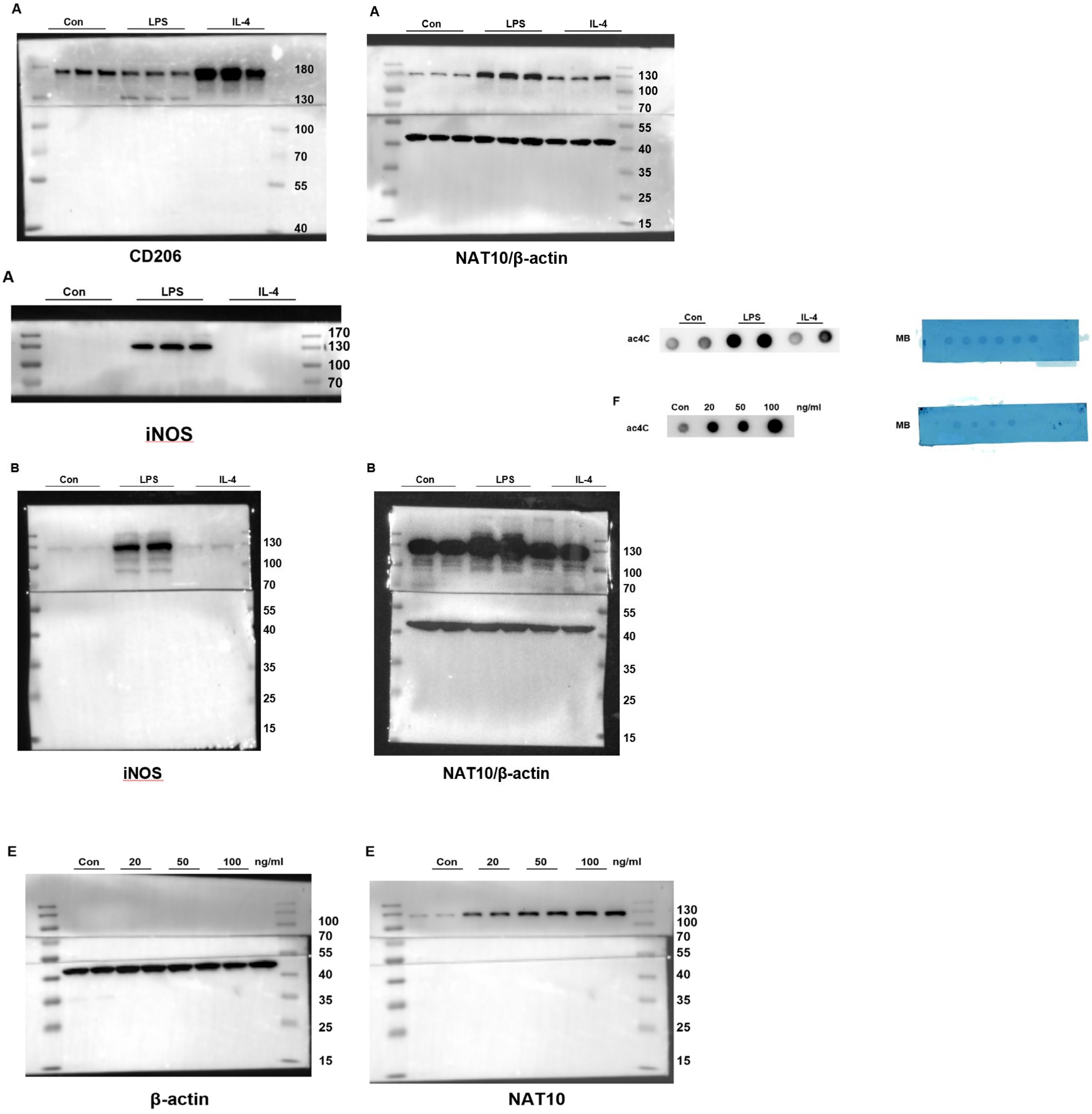
A
Con
LPS
IL-4
180
130
100
70
55
40
CD206
A
Con
LPS
IL-4
130
100
70
55
40
35
25
15
NAT10/β-actin
A
Con
LPS
IL-4
170
130
100
70
iNOS
Con
LPS
IL-4
ac4C
MB
F
Con
20
50
100
ng/ml
ac4C
MB
B
Con
LPS
IL-4
130
100
70
55
40
35
25
15
iNOS
B
Con
LPS
IL-4
130
100
70
55
40
35
25
15
NAT10/β-actin
E
Con
20
50
100
ng/ml
100
70
55
40
35
25
15
β-actin
E
Con
20
50
100
ng/ml
130
100
70
55
40
35
25
15
NAT10

Figure2

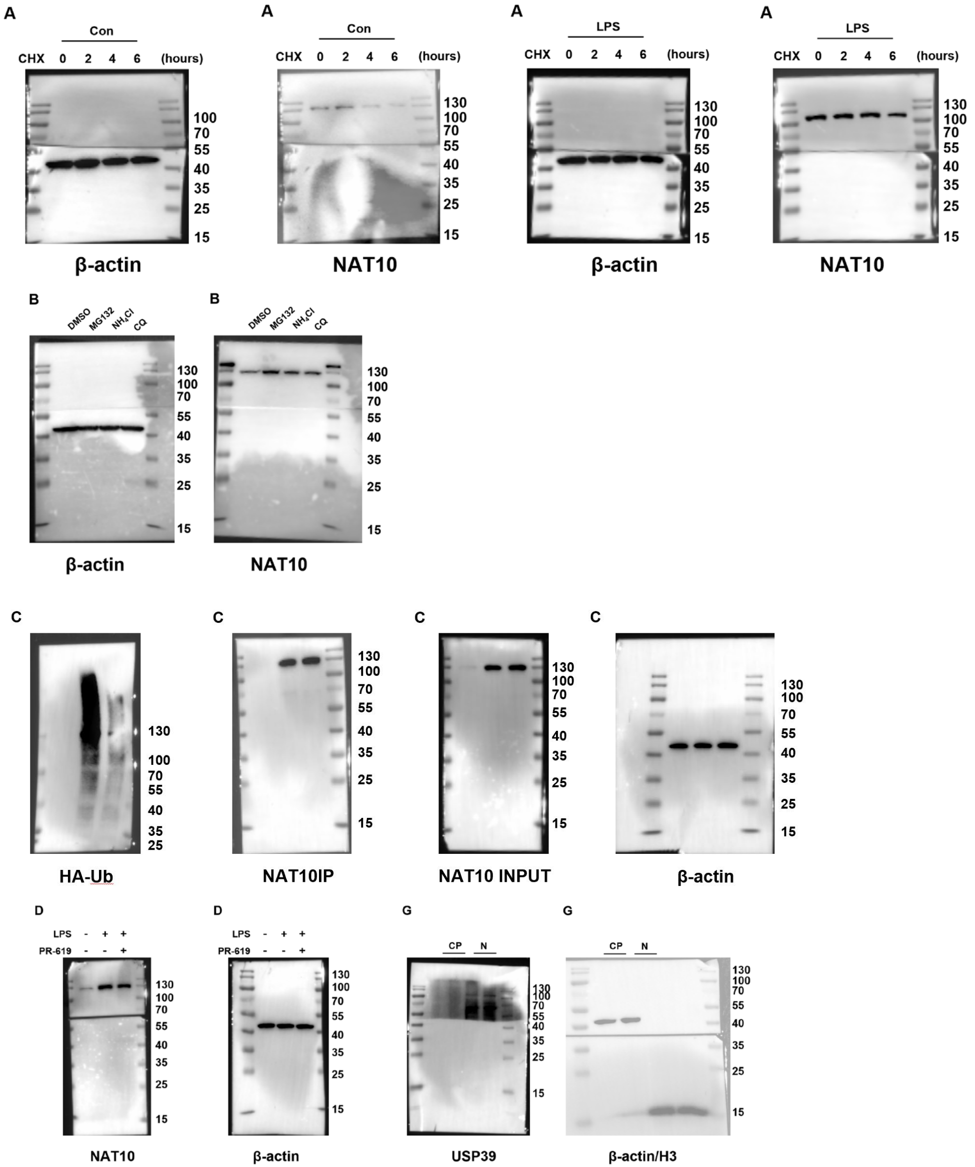
A
Con
CHX 0 2 4 6 (hours)
100
70
55
40
35
25
15
β-actin
A
Con
CHX 0 2 4 6 (hours)
130
100
70
55
40
35
25
15
NAT10
A
LPS
CHX 0 2 4 6 (hours)
130
100
70
55
40
35
25
15
β-actin
A
LPS
CHX 0 2 4 6 (hours)
130
100
70
55
40
35
25
15
NAT10
B
DMSO
MG132
NH4Cl
CQ
130
100
70
55
40
35
25
15
β-actin
B
DMSO
MG132
NH4Cl
CQ
130
100
70
55
40
35
25
15
NAT10
C
130
100
70
55
40
35
25
HA-Ub
C
130
100
70
55
40
35
25
15
NAT10IP
C
130
100
70
55
40
35
25
15
NAT10 INPUT
C
130
100
70
55
40
35
25
15
β-actin
D
LPS - + +
PR-619 - - +
130
100
70
55
40
35
25
15
NAT10
D
LPS - + +
PR-619 - - +
130
100
70
55
40
35
25
15
β-actin
G
CP
N
130
100
70
55
40
35
25
15
USP39
G
CP
N
130
100
70
55
40
35
25
15
β-actin/H3

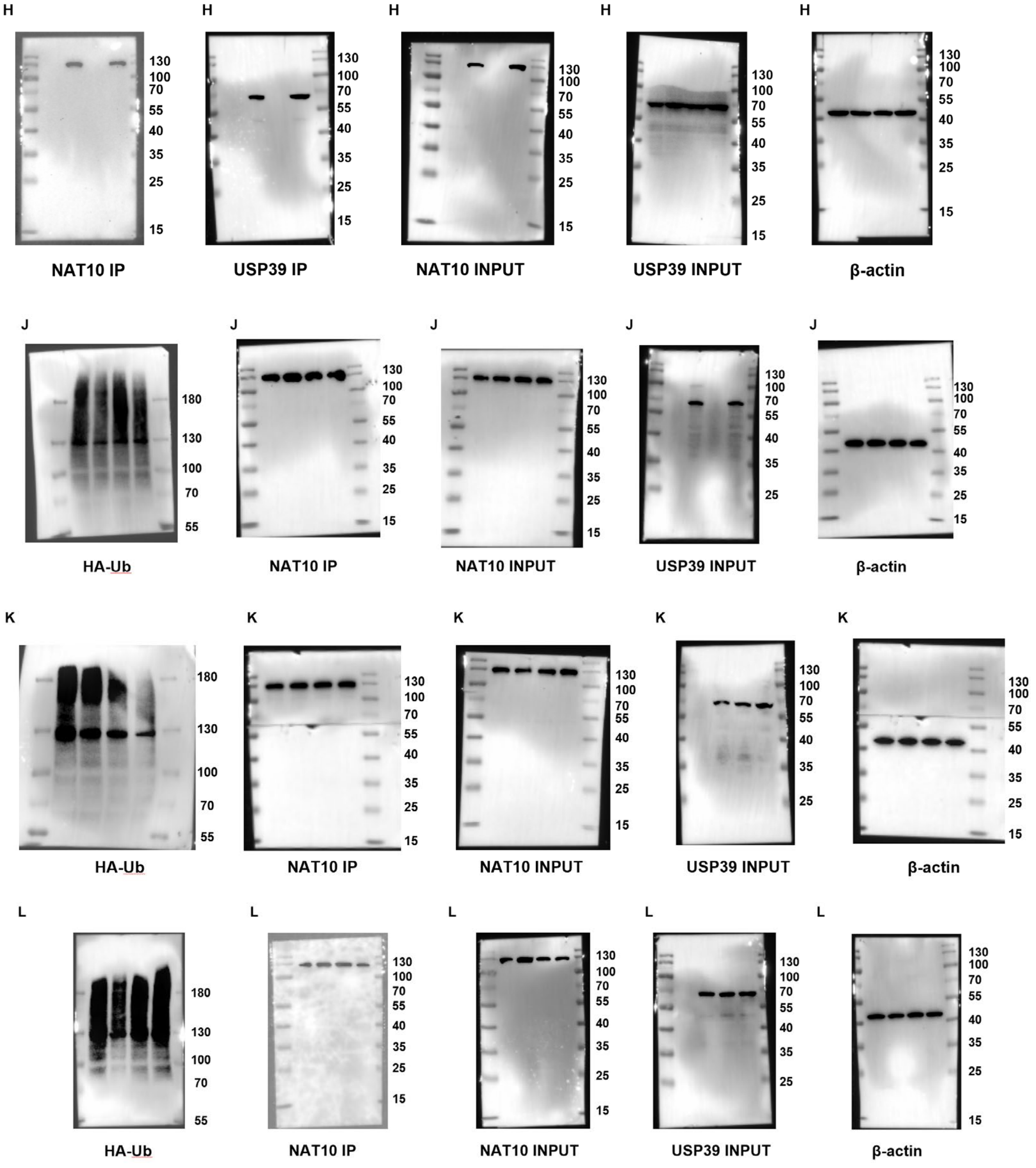
H
NAT10 IP
USP39 IP
NAT10 INPUT
USP39 INPUT
β-actin
J
HA-Ub
NAT10 IP
NAT10 INPUT
USP39 INPUT
β-actin
K
HA-Ub
NAT10 IP
NAT10 INPUT
USP39 INPUT
β-actin
L
HA-Ub
NAT10 IP
NAT10 INPUT
USP39 INPUT
β-actin

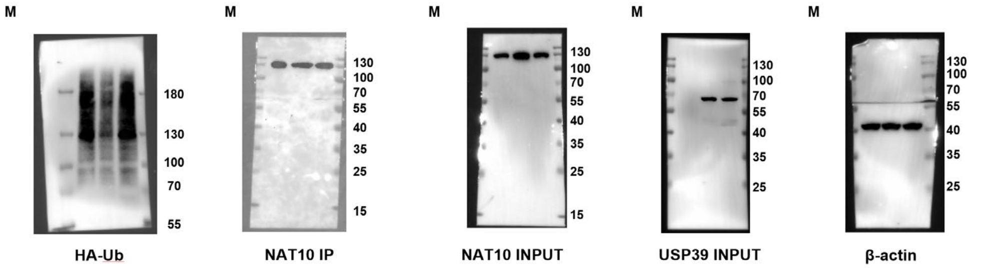
M
180
130
100
70
55
HA-Ub
M
130
100
70
55
40
35
25
15
NAT10 IP
M
130
100
70
55
40
35
25
15
NAT10 INPUT
M
130
100
70
55
40
35
25
USP39 INPUT
M
130
100
70
55
40
35
25
β-actin

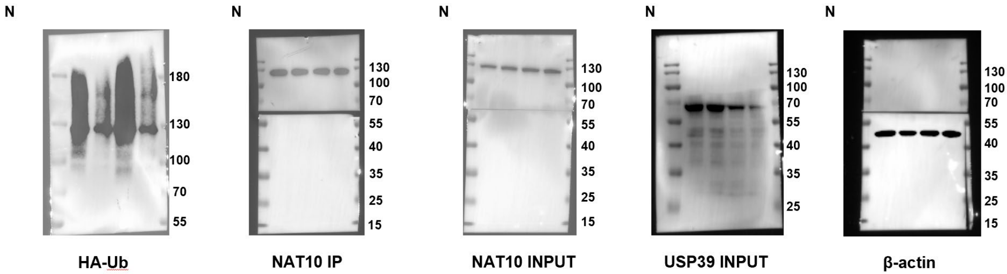
N
180
130
100
70
55
HA-Ub
N
130
100
70
55
40
35
25
15
NAT10 IP
N
130
100
70
55
40
35
25
15
NAT10 INPUT
N
130
100
70
55
40
35
25
USP39 INPUT
N
130
100
70
55
40
35
25
15
β-actin

Figure3

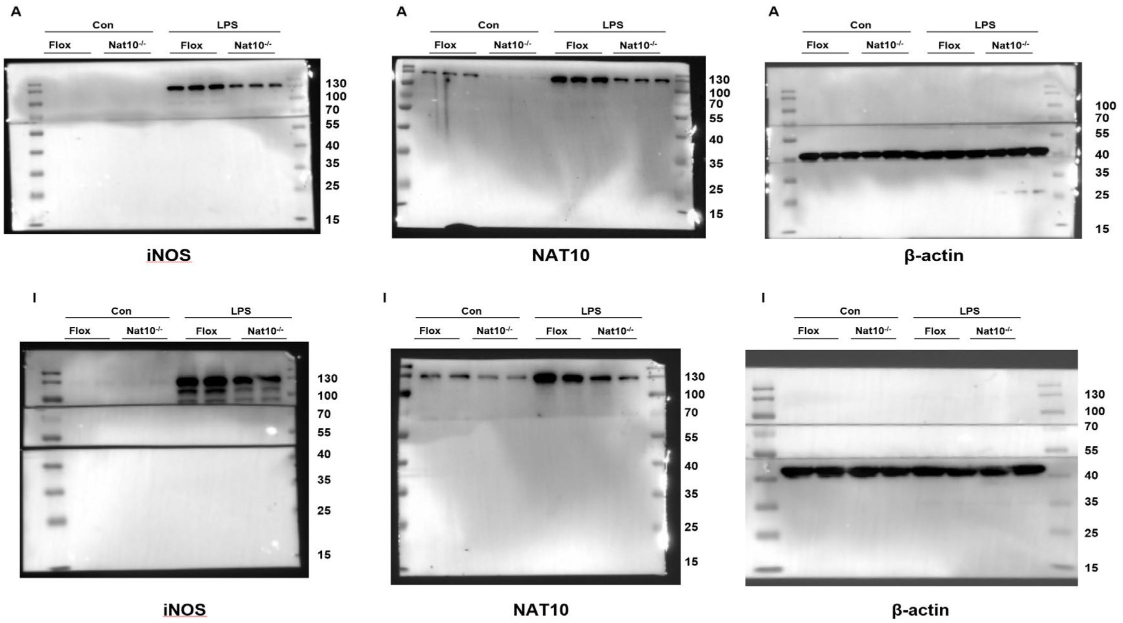
A
Con
LPS
Flox
Nat10-/-
130
100
70
55
40
35
25
15
iNOS
NAT10
β-actin
I

Figure5

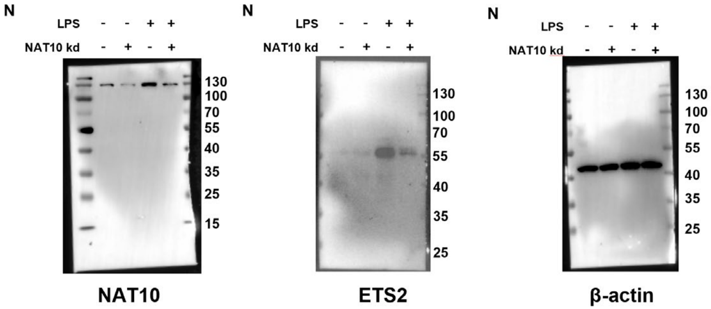
N
LPS - - + +
NAT10 kd - + - +
130
100
70
55
40
35
25
15
NAT10
N
LPS - - + +
NAT10 kd - + - +
130
100
70
55
40
35
25
ETS2
N
LPS - - + +
NAT10 kd - + - +
130
100
70
55
40
35
25
β-actin

Figure6

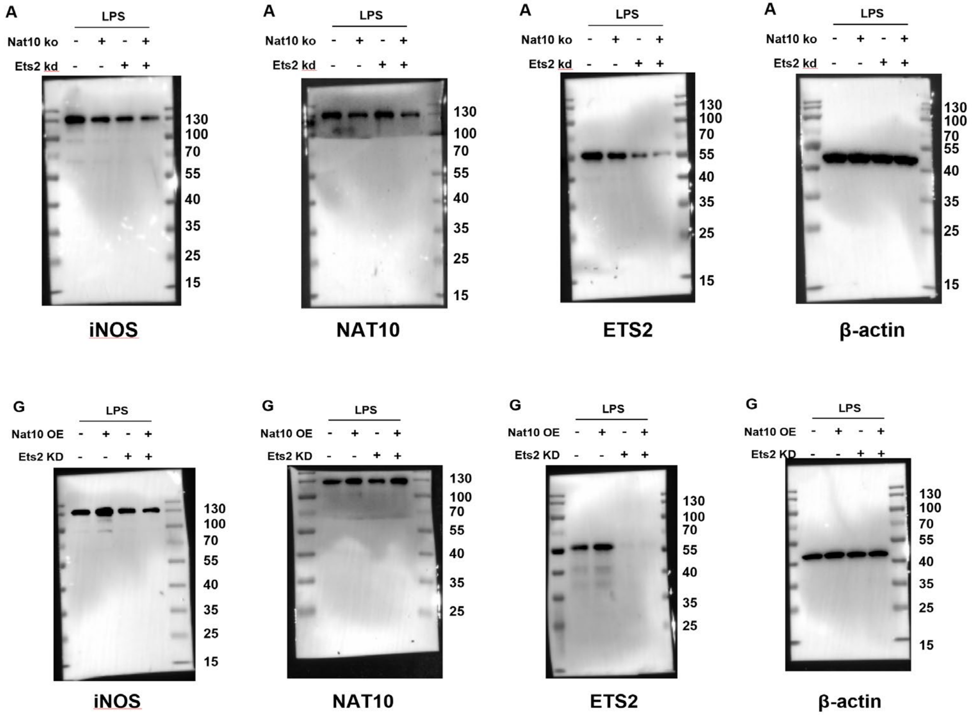
A
LPS
Nat10 ko - + - +
Ets2 kd - - + +
130
100
70
55
40
35
25
15
iNOS
NAT10
ETS2
β-actin
G
LPS
Nat10 OE - + - +
Ets2 KD - - + +
iNOS
NAT10
ETS2
β-actin

FigureS1

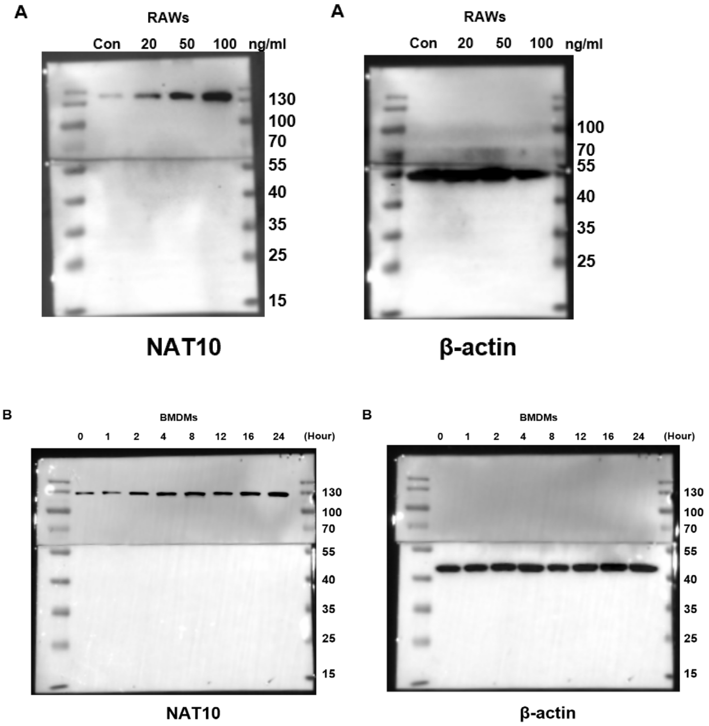
A
RAWs
Con 20 50 100 ng/ml
130
100
70
55
40
35
25
15
NAT10
A
RAWs
Con 20 50 100 ng/ml
100
70
55
40
35
25
β-actin
B
BMDMs
0 1 2 4 8 12 16 24 (Hour)
130
100
70
55
40
35
25
15
NAT10
B
BMDMs
0 1 2 4 8 12 16 24 (Hour)
130
100
70
55
40
35
25
15
β-actin

FigureS2

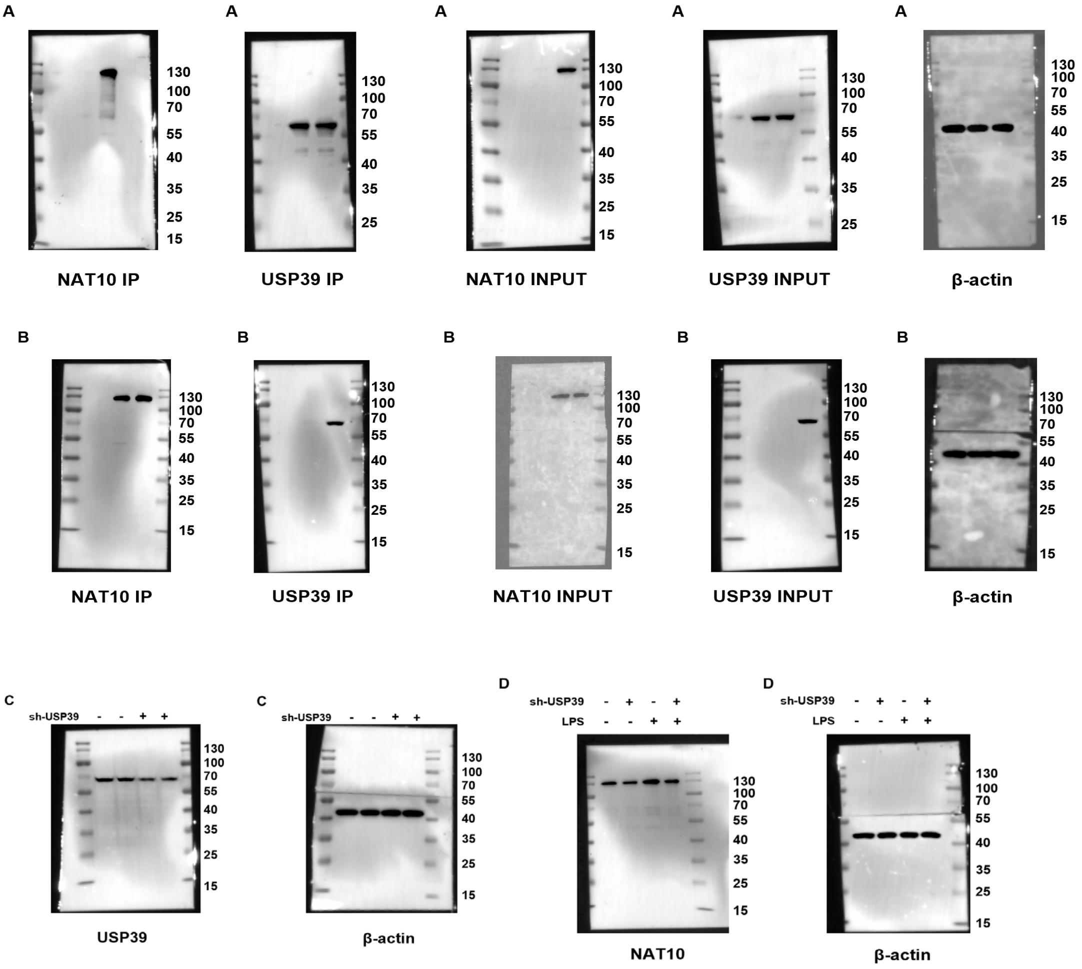

A
130
100
70
55
40
35
25
15
NAT10 IP
USP39 IP
NAT10 INPUT
USP39 INPUT
β-actin
B
NAT10 IP
USP39 IP
NAT10 INPUT
USP39 INPUT
β-actin
C
sh-USP39 - - + +
USP39
β-actin
D
sh-USP39 - + - +
LPS - - + +
NAT10
β-actin

FigureS3

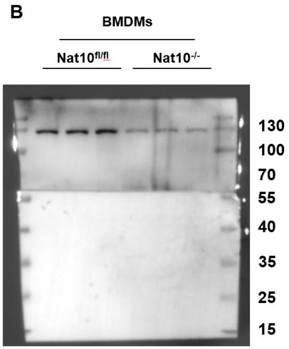

NAT10

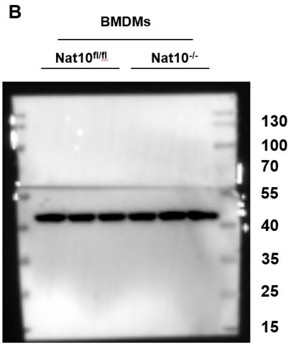

β-actin

FigureS5

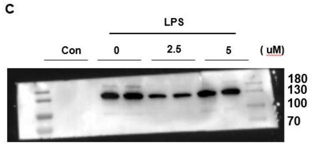

iNOS

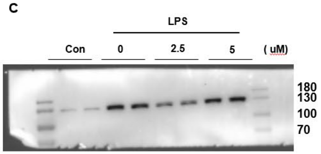

NAT10

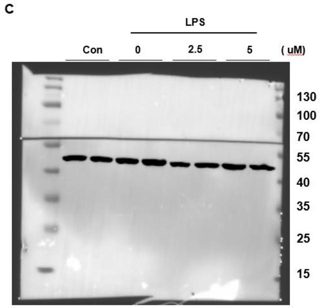

β-actin

FigureS9

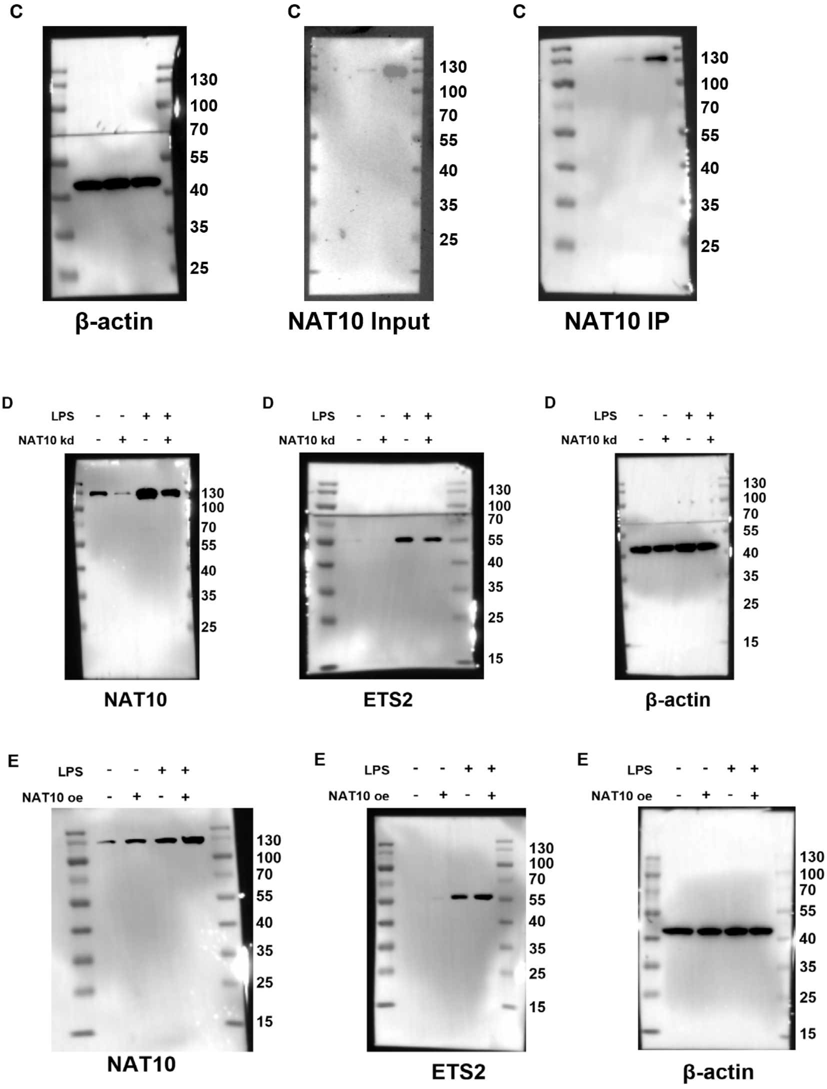
C
β-actin
130
100
70
55
40
35
25
C
NAT10 Input
130
100
70
55
40
35
25
C
NAT10 IP
130
100
70
55
40
35
25
D
LPS - - + +
NAT10 kd - + - +
NAT10
130
100
70
55
40
35
25
D
LPS - - + +
NAT10 kd - + - +
ETS2
130
100
70
55
40
35
25
15
D
LPS - - + +
NAT10 kd - + - +
β-actin
130
100
70
55
40
35
25
15
E
LPS - - + +
NAT10 oe - + - +
NAT10
130
100
70
55
40
35
25
15
E
LPS - - + +
NAT10 oe - + - +
ETS2
130
100
70
55
40
35
25
15
E
LPS - - + +
NAT10 oe - + - +
β-actin
130
100
70
55
40
35
25
15
